# Bacterioferritin: a key iron storage modulator that affects strain growth and butenyl-spinosyn biosynthesis in *Saccharopolyspora pogona*

**DOI:** 10.1186/s12934-021-01651-x

**Published:** 2021-08-14

**Authors:** Jianli Tang, Zirong Zhu, Haocheng He, Zhudong Liu, Ziyuan Xia, Jianming Chen, Jinjuan Hu, Li Cao, Jie Rang, Ling Shuai, Yang Liu, Yunjun Sun, Xuezhi Ding, Shengbiao Hu, Liqiu Xia

**Affiliations:** Hunan Provincial Key Laboratory for Microbial Molecular Biology, State Key Laboratory of Developmental Biology of Freshwater Fish, College of Life Science, Hunan Normal University, Changsha, 410081 China

**Keywords:** *Saccharopolyspora pogona*, Bacterioferritin, Butenyl-spinosyn, Quantitative proteomics, Secondary metabolism

## Abstract

**Background:**

Butenyl-spinosyn, produced by *Saccharopolyspora pogona*, is a promising biopesticide due to excellent insecticidal activity and broad pesticidal spectrum. Bacterioferritin (Bfr, encoded by *bfr*) regulates the storage and utilization of iron, which is essential for the growth and metabolism of microorganisms. However, the effect of Bfr on the growth and butenyl-spinosyn biosynthesis in *S. pogona* has not been explored.

**Results:**

Here, we found that the storage of intracellular iron influenced butenyl-spinosyn biosynthesis and the stress resistance of *S. pogona*, which was regulated by Bfr. The overexpression of *bfr* increased the production of butenyl-spinosyn by 3.14-fold and enhanced the tolerance of *S. pogona* to iron toxicity and oxidative damage, while the knockout of *bfr* had the opposite effects. Based on the quantitative proteomics analysis and experimental verification, the inner mechanism of these phenomena was explored. Overexpression of *bfr* enhanced the iron storage capacity of the strain, which activated polyketide synthase genes and enhanced the supply of acyl-CoA precursors to improve butenyl-spinosyn biosynthesis. In addition, it induced the oxidative stress response to improve the stress resistance of *S. pogona*.

**Conclusion:**

Our work reveals the role of Bfr in increasing the yield of butenyl-spinosyn and enhancing the stress resistance of *S. pogona*, and provides insights into its enhancement on secondary metabolism, which provides a reference for optimizing the production of secondary metabolites in actinomycetes.

**Supplementary Information:**

The online version contains supplementary material available at 10.1186/s12934-021-01651-x.

## Background

Soil microbial actinomycetes can produce a variety of secondary metabolites, many of which are biologically active natural products that have important application value in industry, medicine and agriculture, and are valuable resources for human development and utilization [1–3]. Butenyl-spinosyn, a secondary metabolite produced by the aerobic fermentation of the soil actinomycete *Saccharopolyspora pogona*, is a spinosyn structural analog [4], that effectively kills pests by paralyzing the nervous system of insects [5].This unique insecticidal mechanism makes it powerful in the insecticidal spectrum, harmless to humans and animals, environmentally friendly, safe as a biological pesticide and as a chemical pesticide, giving it broad application prospects in agriculture [6, 7].

The side chain groups of butenyl-spinosyn are easily modified to generate many derivatives, and more than 30 derivatives have been isolated and identified so far [4]. However, the low yield of wild-type butenyl-spinosyn under natural conditions hinders its industrial production and application [8, 9]. Initially, culture media optimization, physical or chemical mutagenesis, and gene rearrangement have been used to explore the potential productivity of secondary metabolites from *Streptomyces* [10, 11]. With the development of genetic manipulation technology and research on biosynthetic pathways and metabolic regulation networks, the use of genetic engineering, metabolic engineering, and construction of chassis cell heterologous biosynthesis has made strain modification easier and more efficient [12, 13].

Preliminary studies have been conducted on the metabolic pathways and regulatory mechanisms of butenyl-spinosyn biosynthesis [14]. Genome sequencing comparison has revealed that the PKS gene in the butenyl-spinosyn biosynthetic gene cluster is very similar to spinosyn (91%–94%), and there are many similarities in their biosynthetic and metabolic pathways [8].The main difference is that of the 5 functional domains encoded by *busA* in the butenyl-spinosyn gene cluster, a butenyl group replaces the ethyl group at position C21 [15, 16]. Short-chain acyl-CoAs, such as acetyl-CoA, malonyl-CoA, methylmalonyl-CoA and propanoyl-CoA, are important precursors for the synthesis of many polyketide secondary metabolites, including butenyl-spinosyn [15, 17]. Previous studies have shown that the *tetR* family of transcriptional regulators play an important regulatory role in the growth and biosynthesis of butenyl-spinosyn in *S. pogona* [18, 19]. Analysis of metabolic pathways, targeted overexpression of key modules in the biosynthetic process or deletion of competitive PKS gene clusters can significantly increase the production of butenyl-spinosyn [8]. Song, et al used RedEx technology to replace different modules based on the gene cluster of spinosyn and performed heterologous expression in *Streptomyces albicans*, successfully synthesizing butenyl-spinosyn [20]. In addition, the concentrations of inorganic salts, such as phosphate and iron salt necessary for microorganisms in culture medium affected the growth and secondary metabolism in *Streptomyces* [21–23].

Iron is a trace element necessary for the growth and development of microorganisms, and it participates in various life activities such as oxygen transport, DNA synthesis, and catalytic protease reactions in cells [24, 25]. However, the extremely low solubility of Fe^3+^ (~ 10^-18^ M) at normal physiological pH limits its utilization in biological cells, and excessive Fe^2+^ will catalyze the production of a large number of reactive oxygen free radicals and cause oxidative damage to cells [26]. Therefore, microorganisms have evolved a way to obtain and store iron through bacterioferritin (Bfr), so that the iron content in cells is strictly maintained within an appropriate range [27, 28]. Bfr can store extracellular free iron in the form of Fe^3+^, for release in the form of Fe^2+^ when required by cell biological metabolic activities [29]. Additionally, Bfr can isolate iron in the cavity when the iron concentration is very high to avoid oxidation by hydrogen peroxide, superoxide, ozone, etc., and assist cells enhancing resistance to oxidative stress [30]. Bfr plays an important role in the growth and development of a variety of bacteria, such as nodulation and nitrogen fixation in *Azorhizobium caulinodans* [31], and could also improve the virulence of *Agrobacterium tumefaciens* by regulating iron homeostasis and oxidative stress [32]. Pathogenesis and drug resistance are also closely related to Bfr in *Mycobacterium tuberculosis* [33]. In addition, inhibiting the BfrB:Bfd interaction in *Pseudomonas aeruginosa* led to the accumulation of Fe^3+^ and the lack of Fe^2+^ in the cytoplasm [34]. Although the functions of Bfr in other bacteria are clear, there are few reports about the effects of Bfr on the growth, development and biosynthesis of secondary metabolites in actinomycetes.

In this study, we found that the concentration of Fe^2+^ in the medium is closely related to the growth and biosynthesis of butenyl-spinosyn in *S. pogona*. Manipulating *bfr* led to a very large difference in the phenotype of the strains and the production of butenyl-spinosyn with altered iron storage capacity. Quantitative proteomics and experimental verification were implemented to gain insight into the metabolic mechanisms of these phenomena. We proposed a working model to explain the effect of Bfr on growth and butenyl-spinosyn biosynthesis in *S. pogona*.

## Results

### Iron affects the growth and biosynthesis of butenyl-spinosyn in S. pogona

**Fig. 1:**
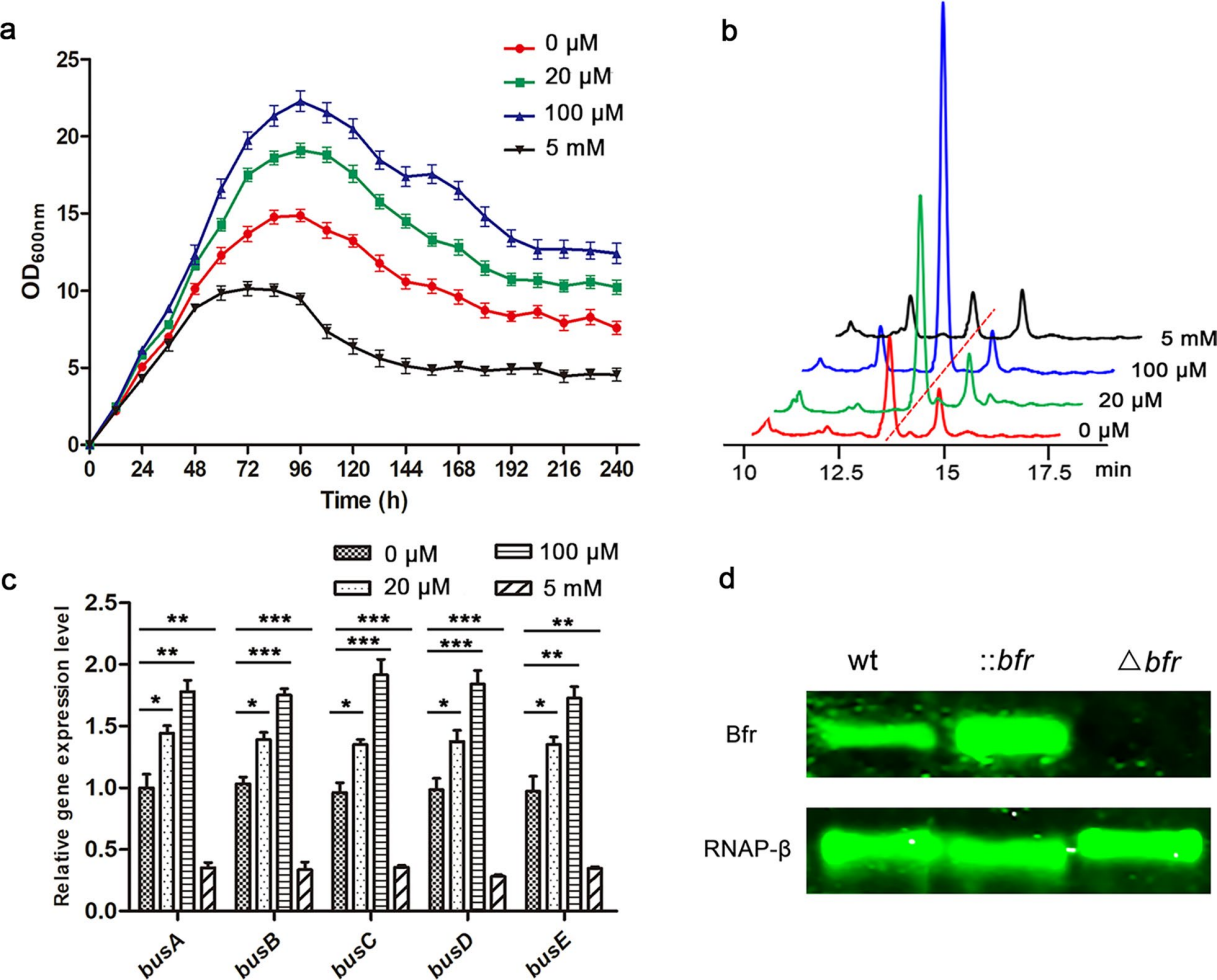
The effects of iron on *S. pogona* and Western blot verification of *bfr* recombinant strains. **a** Effects of different Fe concentrations (0, 20, 100 μM and 5 mM) on strain growth. **b** Effects of different Fe concentrations (0, 20, 100 μM and 5 mM) on the production of butenyl-spinosyn. The detection wavelength was set to 250 nm, and the chromatographic peak of butenyl-spinosyn appeared at approximately 13 min. **c** Effects of different Fe concentrations (0, 20, 100 μM and 5 mM) on the gene transcriptional levels of polyketide synthase genes. *S. pogona* was cultured in SFM containing different concentrations of Fe at 30 °C for 4 d, and total RNA was extracted separately and analyzed by qRT-PCR. 16S rRNA served as the normalization control. **d** Western blot verification of *bfr* recombinant strains. RNAP-β was used as an internal protein control. The averages from three biological replicates are shown. Error bars represent the standard deviation of the mean. *, ** and *** indicate P < 0.05, P < 0.01 and P < 0.005, respectively, compared to the wild-type under the same conditions

To explore the effects of iron on growth and secondary metabolism in *S. pogona*, different concentrations of FeSO_4_ (0 μM, 20 μM, 100 μM and 5 mM) were added into SFM, the strain density of *S. pogona* and butenyl-spinosyn production were subsequently measured. It clearly showed that the strain density elevated with increasing iron concentration within a suitable range (0–100 μM), but excessive iron (5 mM) significantly inhibited the growth of the strain and caused the stable growth period to advance and enter the decline phase prematurely (Fig. 1a).

High performance liquid chromatography (HPLC) was used to detect the *S. pogona* fermentation broth extract samples to explore the production of butenyl-spinosyn in different iron concentration (Fig. 1b). There was a significant difference in the HPLC peak at approximately 13 min, which was confirmed to be butenyl-spinosyn by LC-MS/MS analysis (Additional file 1: Figure S1). The yield of butenyl-spinosyn reached the maximum when the iron concentration was 100 μM, and the lowest when the iron concentration was 5 mM. The qRT-PCR checking of the transcriptional levels of polyketide synthase genes (*busA*, *busB*, *busC*, *busD*, *busE*) , which control the synthesis of butenyl-spinosyn carbon skeleton [15], was consistent with that of HPLC analysis (Fig. 1c). The results showed that the biosynthesis of butenyl-spinosyn was closely related to the iron concentration in the medium, and an appropriate iron concentration was beneficial for the synthesis of butenyl-spinosyn. The above results strongly suggested that iron played an important role in the growth, development and biosynthesis of butenyl-spinosyn in *S. pogona*.

An orf (orf 11096-6924, termed as *bfr* thereafter) which encodes an analogue of bacterioferritin (Bfr) was found by analyzing the whole-genome of *S. pogona*. Phylogenetic tree analysis showed that Bfr is widespread in actinomycetes (Additional file 1: Figure S2). In order to explore whether *bfr* affects the iron storage, growth and biosynthesis of butenyl-spinosyn in *S. pogona*, *bfr* knockout strain (Δ*bfr*) and overexpression strain (::*bfr*) were subsequently constructed (Additional file 1: Figure S3) and verified by using PCR, qRT-PCR and Western blot (Additional file 1: Figure S4, Fig. 1d).

### bfr affects the iron storage capacity and growth of S. pogona

**Fig. 2:**
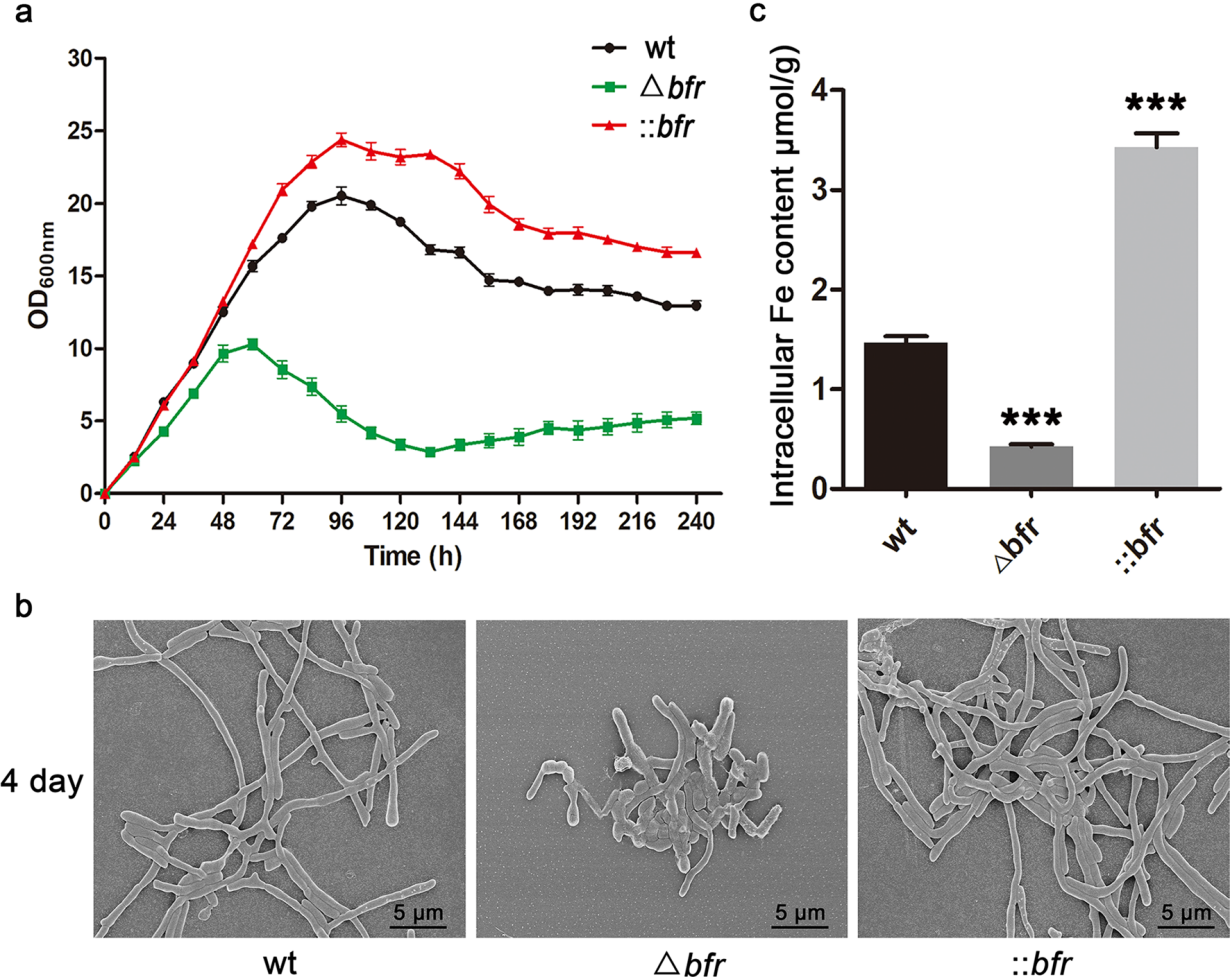
Effects of *bfr* mutation on strain growth and iron storage capacity. **a** Comparison of the growth of the wild-type and mutant strains in SFM with 100 μM Fe. **b** SEM observations of the morphological differences between the wild-type and mutants on the fourth day. **c** Determination of the total iron content in the wild-type and the mutant strains. The averages from three biological replicates are shown. Error bars represent the standard deviation of the mean. *, ** and *** indicate P < 0.05, P < 0.01 and P < 0.005, respectively, compared to the wild-type under the same conditions

Taking into account the influence of different iron concentrations on the growth of *S. pogona* (Fig. 1a), the strain density in SFM containing 100 μM FeSO_4_ were measured (Fig. 2a). Compared to the wild-type, ::*bfr* was more vigorous, having a longer stable period and the largest strain density, while the growth of Δ*bfr* was significantly inhibited, and the stable period was advanced, which was similar to the original strain under the inhibition of excess iron (5 mM) (Fig. 1a). Scanning electron microscopy (SEM) was used to observe morphology of *S. pogona* (Fig. 2b). The wild-type and ::*bfr* strains had normal growth morphology, while the hyphae of Δ*bfr* were short and broken, indicating that Δ*bfr* had entered the decline period prematurely, which was consistent with the growth curve trend. These results indicated that *bfr* could affect the iron storage potential of *S. pogona*.

Subsequently, the intracellular iron content was measured to verify the effects of Bfr on the iron storage capacity of *S. pogona* (Fig. 2c). The intracellular iron concentration of ::*bfr* was 3.42 ± 0.24 μmol/g, which was significantly higher than that of the wild-type (1.50 ± 0.11 μmol/g), while the intracellular iron concentration of Δ*bfr* was only 0.42 ± 0.04 μmol/g. The results showed that the iron storage capacity of ::*bfr* was significantly stronger than that of Δ*bfr* and the wild-type strains, confirming that the difference in iron storage capacity caused by the mutation of *bfr* was an important reason for the difference in the growth of *S. pogona*.

### Overexpression of bfr enhances iron toxicity tolerance and delays sporulation

**Fig. 3:**
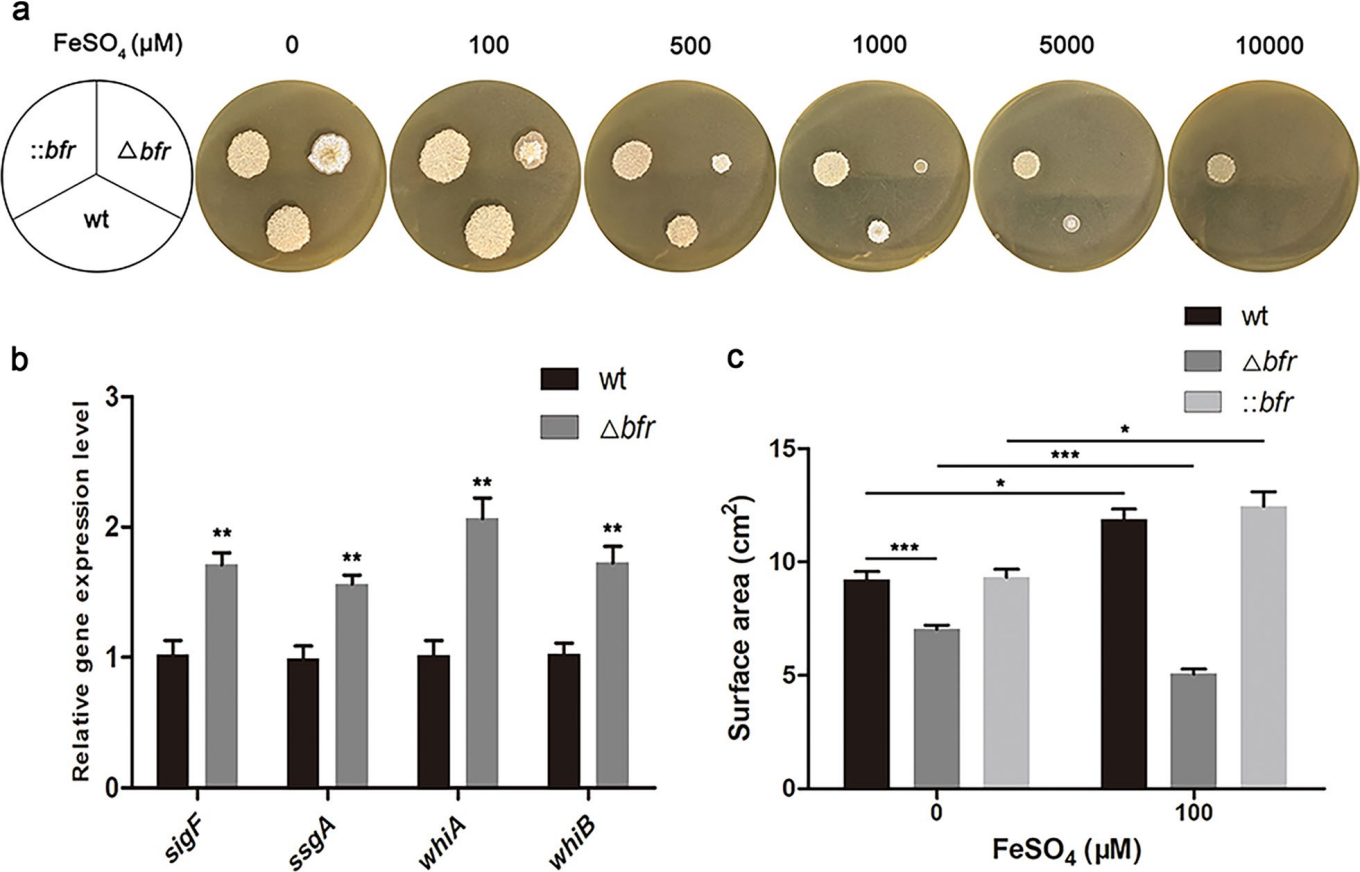
The differences in growth and sporulation between the wild-type and mutants on CSM medium. **a** The difference in growth of the mutants containing different FeSO_4_ concentrations (0, 100, 500, 1000, 5000, and 10,000 μM). The strains were inoculated with 5 μL on each CSM plate. **b** qRT-PCR analysis of the expression levels of spore-related genes. Total RNA of the wild-type and Δ*bfr* strains was extracted separately on the fourth day. The 16S rRNA served as the normalization control. **c** Colony surface area statistics of the wild-type and mutant strains. Averages from three biological replicates are shown. Error bars represent the standard deviation of the mean. *, **and *** indicate P < 0.05, P < 0.01 and P < 0.005, respectively, compared to the wild-type under the same conditions

To explore the role of Bfr in strains resisting high concentrations of iron, we added different concentration gradients of FeSO_4_ (0, 100, 500, 1000, 5000, and 10,000 μM) to the CSM solid medium followed by cultivation for 6 days, then monitor the growth of *S. pogona* (Fig. 3a). Without supplemental of FeSO_4_ in CSM, the timing of sporulation was advanced and the yield of spores was elevated in Δ*bfr* compared with the wild-type and ::*bfr*. The qRT-PCR results showed that the transcription levels of the sporulation-related genes (*sigF*, *ssgA*, *whiA*, *whiB*) in Δ*bfr* were significantly higher than those in the wild-type strain, which increased by 1.68-fold, 1.58-fold, 2.04-fold and 1.68-fold, respectively (Fig. 3b). These results indicated that the deletion of *bfr* promoted the expression of sporulation genes and improved sporulation ability.

The colony surface area statistics on CSM with 0 and 100 μM FeSO_4_ showed that the growth rate of Δ*bfr* colonies was significantly lower than that of the wild-type and ::*bfr* colonies, which was consistent with the results of the growth curve in SFM liquid culture (Fig. 3c). Notably, the colony surface area of the wild-type and ::*bfr* strains with 100 μM FeSO_4_ was significantly larger than that without FeSO_4_, while the result for Δ*bfr* was the opposite, further showing that the growth of Δ*bfr* was highly sensitive to the toxicity of Fe^2+^. As the concentration of FeSO_4_ continued to increase, the growth of the three strains was gradually inhibited to varying degrees. The Δ*bfr* could hardly grow when the concentration of FeSO_4_ was above 1000 μM, while the wild-type strain could grow and with the sporulation ahead of schedule, which may be due to feedback from the high iron concentration stress. The wild-type strain could hardly grow at a concentration of 5000 μM FeSO_4_, while ::*bfr* could, although compared with the strain without FeSO_4_, the surface area of the ::*bfr* colonies was slightly reduced at the highest iron level, and spores were hardly produced early. These results suggested that the overexpression of *bfr* enhanced the tolerance to iron toxicity and hindered sporulation in *S. pogona*.

### Effects of bfr on butenyl-spinosyn biosynthesis and insecticidal toxicity

**Fig. 4:**
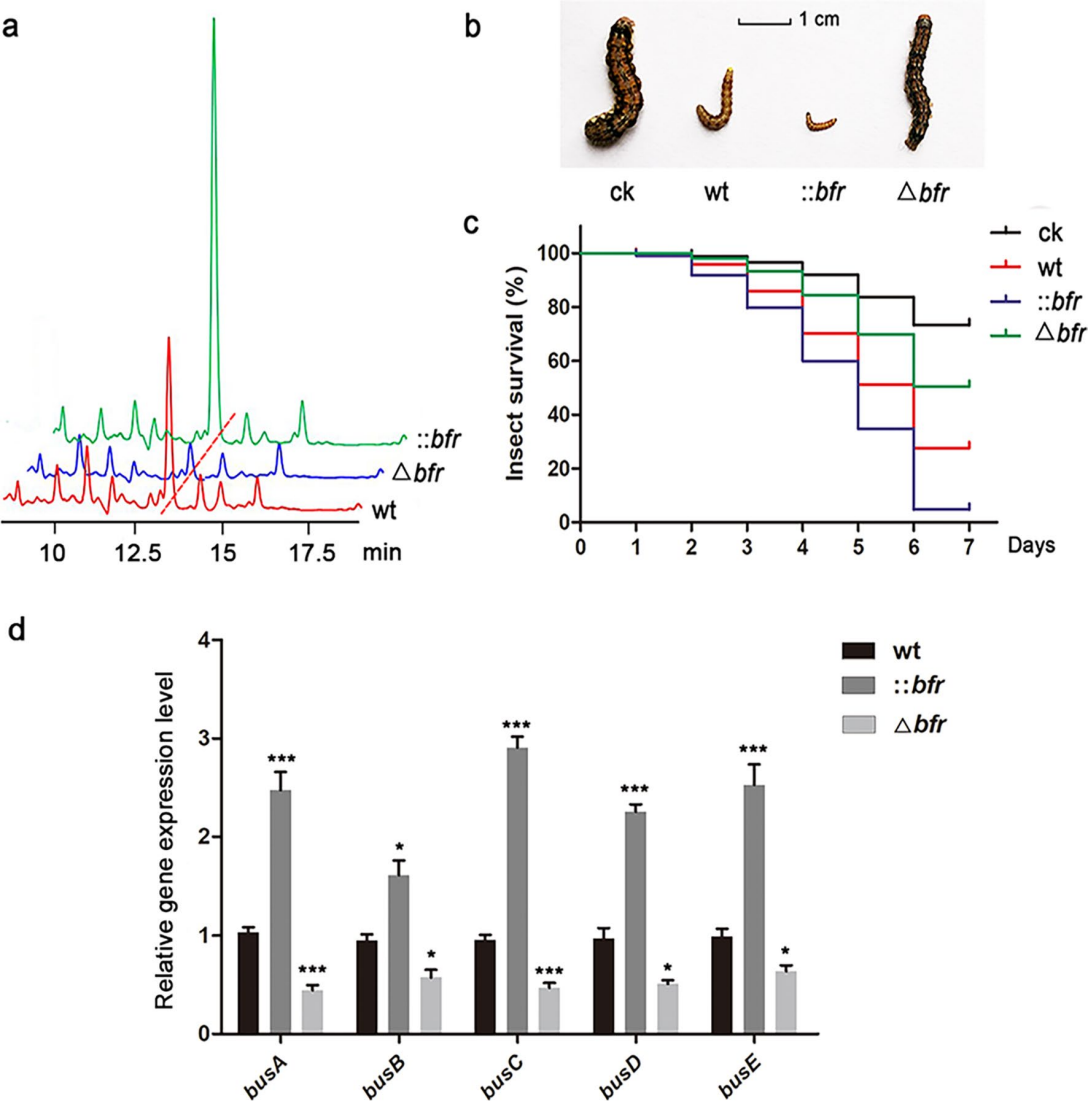
Yield detection of butenyl-spinosyn and expression levels analysis of polyketide synthase genes. **a** HPLC detection of the production of butenyl-spinosyn in the wild-type and the mutant strains. **b** Morphological differences of the surviving insects. **c** The difference in insecticidal toxicity of the fermentation broth supernatant between the wild-type and mutant strains. **d** qRT-PCR analysis of the expression levels of polyketide synthase genes in the wild-type and mutant strains. The transcriptional levels of these genes (*busA*, *busB*, *busC*, *busD*, *busE*) in ::*bfr* were 2.40-, 1.69-, 3.05-, 2.33-, and 2.55-fold higher than those in the wild-type, respectively. The transcriptional levels of them in Δ*bfr* were 0.43, 0.60, 0.49, 0.53, and 0.64 times that of the wild-type, respectively. 16S rRNA served as the normalization control. Averages from three biological replicates are shown. Error bars represent the standard deviation of the mean. *, **and *** indicate P < 0.05, P < 0.01 and P < 0.005, respectively, compared to the wild-type under the same conditions

The phenotypic differences between the wild-type and mutants were suggestive of whether there were significant differences in butenyl-spinosyn. The HPLC analysis showed that overexpression of *bfr* greatly increased the yield of butenyl-spinosyn (71.22 ± 7.89 mg/L), which was 3.14-fold higher than that of the wild-type (22.65 ± 0.95 mg/L). The yield of butenyl-spinosyn after knocking out *bfr* was significantly reduced (6.43 ± 0.56 mg/L) and only 0.28-fold that of the wild-type (Fig. 4a, Additional file 1: Figure S6).

The difference of butenyl-spinosyn yield between the wild-type and mutants was visually presented in the insecticidal activity against *Helicoverpa armigera* (Fig. 4b, c). The Δ*bfr* mutant showed a slow decline in survival, while the ::*bfr* mutant caused remarkably rapid death, with the lethal time (LT_50_) advancing by 2.111 days (Additional file 1: Table S3), and the surviving insects refused to feed and their growth slowed down.

To explore the effect of *bfr* on butenyl-spinosyn biosynthesis, the expression levels of polyketide synthase genes from *bus* gene cluster were detected by qRT-PCR. The data showed that the expression levels of these genes were upregulated to varying degrees in ::*bfr*, while that were significantly suppressed in Δ*bfr* (Fig. 4d). These results confirmed that *bfr* positively affect the production of butenyl-spinosyn and insecticidal activity of the fermentation broth supernatant by strengthening polyketide synthases in *S. pogona*.

### Quantitative proteomic analysis of S. pogona and the mutant strains

**Fig. 5:**
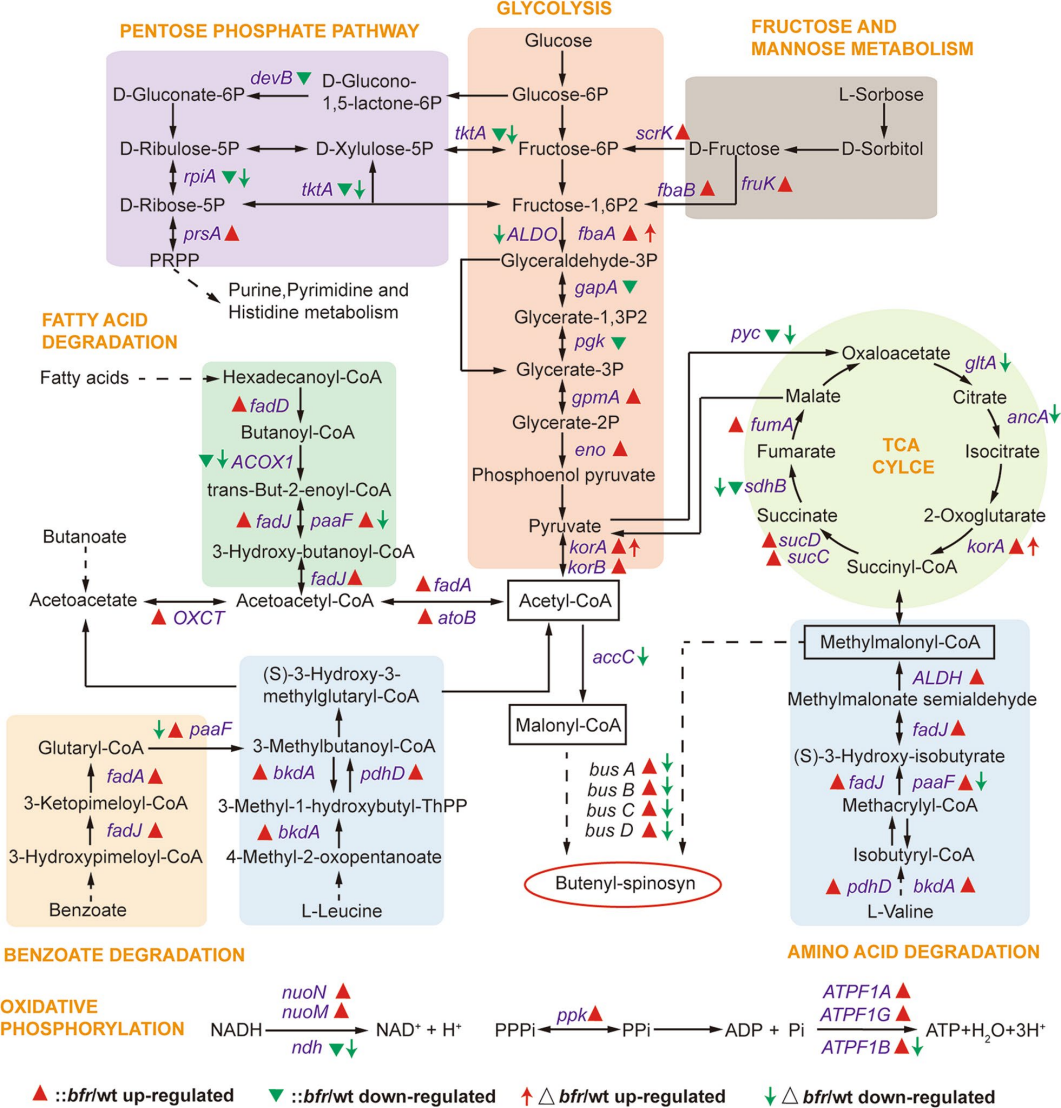
The effects of *bfr* mutation on the acyl-CoA pools and energy-related metabolic pathways. The metabolic network pathway diagram was constructed based on quantitative proteomics data and KEGG analysis. The more DEPs in the *bfr* mutants were those involved in central carbon metabolism, β-oxidation, amino acid degradation and oxidative phosphorylation pathways. Short-chain acyl-CoA pools are framed by rectangles

To further reveal the internal factors underlying the large differences between wild-type and mutant strains, a quantitative proteomics analysis was performed using TMT labeling. A total of 5013 proteins were identified in the wild-type and mutant strains, 428 proteins were upregulated and 536 proteins were downregulated in ::*bfr* and 149 proteins were upregulated and 263 proteins were downregulated in Δ*bfr* (Additional file 1: Figure S7). Based on quantitative proteomics data, a metabolic network pathway diagram was constructed by KEGG analysis (Fig. 5; Additional file 2: Table S4; Additional file 3: Table S5). Compared with the wild-type, the more differentially expressed proteins (DEPs) in the *bfr* mutants were those involved in central carbon metabolism and oxidative phosphorylation pathways.

### Increased acyl-CoA metabolic flux provides more precursor substances for the biosynthesis of butenyl-spinosyn

Acetyl-CoA, methylmalonyl-CoA, malonyl-CoA and other short-chain acyl-CoAs are important sources of precursor substances for primary metabolism and the biosynthesis of butene-spinosyn. The expression levels of rate-limiting enzymes involved in glycolysis (such as *fbaA*, *gpmA*, *eno*, *korA*, and *korB*), fructose and mannose metabolism (such as *scrK*, *fruK* and *fbaB*), fatty acid degradation (such as *fadD*, *fadJ*, *paaF*, *fadA*, and *atoB*), amino acid degradation (such as *bkdA*, *pdhD*, *paaF*, *fadJ*, and *ALDH*), and benzoate degradation (such as *fadA*, *fadJ*, and *paaF*) were all significantly upregulated in ::*bfr* compared to the wild-type strain, which promoted the synthesis of acyl-CoA precursors. Conversely, the expression levels of enzymes involved in the pentose phosphate pathway (such as *devB*, *tktA*, and *rpiA*) and the key rate-limiting enzyme pyruvate carboxylase (*pyc*) that catalyzes the production of oxaloacetate from pyruvate to the TCA cycle were all significantly downregulated, reducing the loss of acetyl-CoA to other pathways. Normal growth of ::*bfr* was ensured due to the upregulation of the key enzymes (such as *korA*, *sucC*, *sucD*, and *fumA*) in the TCA cycle. Moreover, the expression levels of related enzymes involved in the oxidative phosphorylation pathway were significantly upregulated, including the NADH-quinone oxidoreductase subunit (*nuoN*, *nuoM*), polyphosphate kinase (*ppk*) and F-type H^+^-transporting ATPase (*ATPF1A*, *ATPF1B*, *ATPF1G*), providing sufficient energy for cell metabolism [35, 36]. In addition, the four polyketide synthases (BusA, BusB, BusC, BusD) detected were also upregulated to varying degrees in ::*bfr*, which was consistent with the results of qRT-PCR (Additional file 1: Figure S8).

**Fig. 6:**
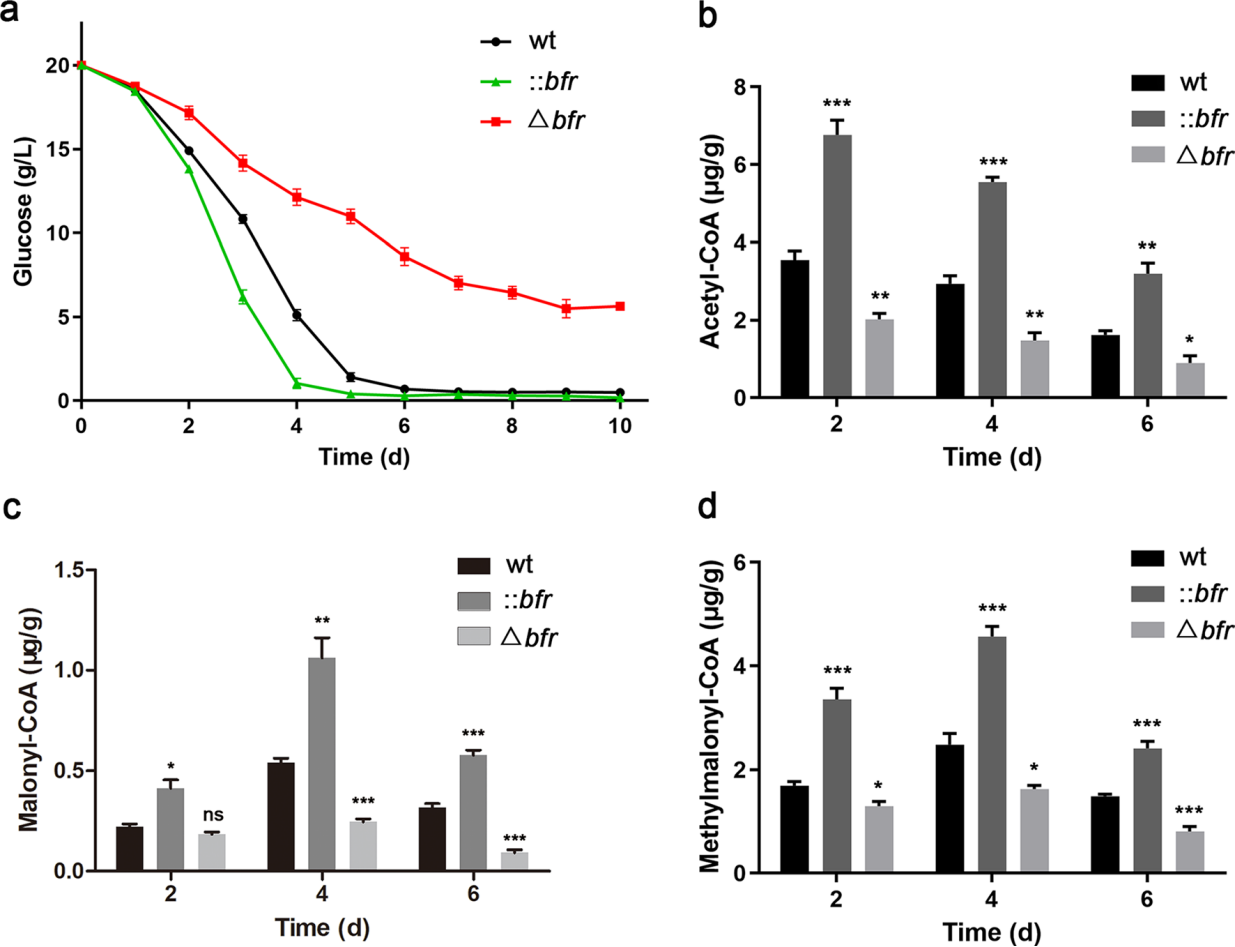
The analysis of glucose consumption and acyl-CoA precursors accumulation.** a** Comparison of glucose utilization of the wild-type and mutants in SFM. Compared with the wild-type, ::*bfr* showed stronger glucose utilization capacity, while the glucose utilization of Δ*bfr* decreased significantly. **b** Determination of acetyl-CoA accumulation in the wild-type and mutants in SFM. **c** Determination of malonyl-CoA accumulation in the wild-type and mutants in SFM. **d** Determination of methylmalonyl-CoA accumulation in the wild-type and mutants in SFM. Averages from three biological replicates are shown. Error bars represent the standard deviation of the mean. *, **and *** indicate P < 0.05, P < 0.01 and P < 0.005, respectively; ns, no significance, compared to the wild-type under the same conditions

The above analysis showed that *bfr* has a positive effect on the expression of enzymes related to central carbon metabolism. The advantage of ::*bfr* in glucose utilization efficiency was confirmed by monitoring the glucose consumption during the fermentation of *S. pogona* (Fig. 6a). Therefore, we speculated that the overexpression of *bfr* could promote the synthesis and accumulation of acetyl-CoA, malonyl-CoA, and methylmalonyl-CoA, providing sufficient precursors for butenyl-spinosyn biosynthesis. The intracellular content of these precursors at different time points was analyzed and the results showed that their concentrations were significantly higher in ::*bfr* than that in the wild-type, confirming our hypothesis (Fig. 6b, c, d).

### Oxidative stress response enhances the stress resistance of ::bfr

The storage and utilization of iron by Bfr in cells generates large amounts of reactive oxygen species (ROS), including superoxide, H_2_O_2_ and hydroxyl radical, which can result to oxidative damage of cells if not scavenged in time [27]. To uncover the reasons for the differences in iron storage and tolerance in *bfr* mutant and wild type strains, we analyzed DEPs associated with iron homeostasis and oxidative stress (Table 1; Additional file 1: Figure S9).

**Table 1.** Proteins differential expression related to iron homeostasis and oxidative stress in *bfr* mutant strains

Accession	Gene	Protein	Possible function	Fold change	
				::*bfr*/wt	Δ*bfr*/wt
A0A2N3XSC8	*A8926_1113* (*bfr*)	Bacterioferritin	Iron-storage protein	2.05	/
A0A2N3XUS8	*A8926_2054* (*fer*)	Ferredoxin	Iron ion binding, electron transfer activity	1.52	0.61
A0A6H1R6Z6	*trxB*	Thioredoxin reductase	Removal of superoxide radicals	1.49	0.55
A0A6H1R2G0	*sodN*	Superoxide dismutase	Superoxide dismutase activity	1.34	0.62
A0A6H1RAW2	*katG*	Catalase-peroxidase	Decomposition of hydrogen peroxide	1.37	0.73

/ indicates that the protein was not detected in the biological repeat

**Fig. 7:**
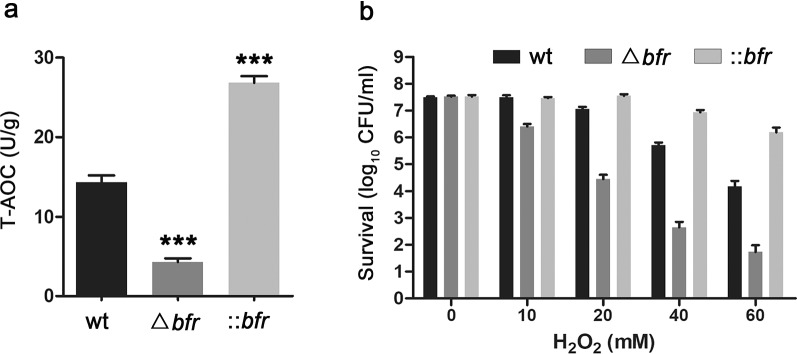
Antioxidant capacity analysis. **a** Total antioxidant capacity (T-AOC) detection. ::*bfr* showed stronger antioxidant capacity than the wild-type. **b** Survival of the wild-type, ::*bfr* and Δ*bfr* strains after exposure to H_2_O_2_. Averages from three biological replicates are shown. Error bars represent the standard deviation of the mean. *, **and *** indicate P < 0.05, P < 0.01 and P < 0.005, respectively, compared to the wild-type under the same conditions

As expected, the expression level of Bfr was significantly higher in ::*bfr* than in the wild-type, and it failed to be detected in Δ*bfr*. In addition, the expression level of ferredoxin, which is involved in cellular iron binding and electron transfer in redox reactions [37], was also significantly higher in ::*bfr*. Notably, the expression levels of thioredoxin reductase (TrxB), catalase-peroxidase (KatG) and superoxide dismutase (SOD), which are involved in oxidative stress response [38–40], were all significantly increased in ::*bfr*, suggesting a possible difference in antioxidant capacity between the mutant and wild-type strains (Table 1). Therefore, the total antioxidant capacity (T-AOC) was detected in different strain, and the result showed that the T-AOC of ::*bfr* was stronger than that of the wild-type, whereas it decreased significantly after deleting *bfr* (Fig. 7a). Furthermore, experimental data demonstrated that ::*bfr* was much more tolerant than wild type strain to H_2_O_2_ exposure (Fig. 7b). These results indicated that the overexpression of *bfr* induce oxidative stress response and thus enhance the strain’s resistance to stress.

## Discussion

Metal ions are essential for the growth and metabolism of microorganisms, as they are often important cofactors for various enzymatic reactions in cells [41–43]. For example, iron is widely involved in various key processes on which life activities depend, such as redox reactions, DNA synthesis, and enzyme catalysis [44, 45]. Microbial cells rely on Bfr to maintain the iron concentration in a relatively stable range. However, due to the extremely low solubility of Fe^3+^ and the toxic ROS produced by Fe^2+^ during the oxidation process, the use of iron by microorganisms is facing a great challenge [24, 46]. The method of improving secondary metabolites by optimizing only the trace elements added to the fermentation medium has great limitations due to the toxicity of an excess of metal ions to cells. Exploring the mechanism by which iron affect the growth of microorganisms and the synthesis of secondary metabolites is of great significance for increasing the production of valuable secondary metabolites. Therefore, the significant influence of iron on the growth and butenyl- spinosyn biosynthesis of *S. pogona* has aroused our interest (Fig. 1).

Bfr plays an important role in regulating iron storage and utilization in microorganisms [28]. It has also been found to be widely present in actinomycetes (Additional file 1: Figure S2). We effectively improved the ability to store and utilize iron by increasing the expression of the Bfr protein in *S. pogona*. Phenotypic experiments of the mutant strains showed that ::*bfr* grows better than the wild-type strain and has stronger anti-iron toxicity. *bfr* knockout in *S. pogona* leads to a sharp decline in the ability to store iron, low strain density, extreme sensitivity to iron toxicity, and induction of the expression of spore-related genes, resulting in a large number of spores (Fig. 2, 3). The premature production of spores is often the response of strains to adversity [47, 48], indicating that the deletion of *bfr* reduced the strain’s ability to adapt to the environment.

Quantitative proteomics analysis showed that ::*bfr* strain has the advantages in central carbon metabolism and energy metabolism compared with the wild-type, which provides a better guarantee for growth (Fig. 5). In addition, ferredoxin is a Fe-S protein that mediates electron transfer in a variety of metabolic reactions, aid in iron storage and utilization, and facilitate related metabolism in ::*bfr* [37]. Fe^2+^ is oxidized to Fe^3+^ and stored in Bfr, and then reduced to Fe^2+^ when needed. Excess iron will aggravate the oxidation-reduction reaction, and a large amount of ROS will be generated in the process [27]. Therefore, the tolerance of strains to iron is closely related to oxidative stress. TrxB can form an antioxidant system with Txr/NADPH to eliminate superoxide [38]. SOD is a kind of antioxidant metal enzymes, which can catalyze the disproportionation of superoxide anion radicals to generate O_2_ and H_2_O_2_, while KatG can decompose H_2_O_2_ to relieve the threat of oxidative damage to cells [39, 49]. The catalytic activity of these enzymes, such as ferredoxin and SOD, often requires the participation of iron [40]. Therefore, the enhancement of iron storage capacity promoted their expression and activity in ::*bfr*. Our results suggested that overexpression of *bfr* leads to an enhanced oxidative stress response, which protects cells from oxidative damage and enhances survival ability of *S. pogona* in an environment with high iron concentration. The enhanced stress resistance of high-yielding strains is very beneficial to the stability of large-scale industrial fermentation in complex environments.

The overexpression of *bfr* also led to a substantial increase in the production of butenyl-spinosyn, which mainly attributed to the activation of polyketide synthase genes and the supply of acyl-CoA flow [50]. The effect of iron on the growth and secondary metabolism of many microorganisms has been revealed by previous studies [22, 23, 28]. In our results, the proper iron concentration not only promoted the growth of *S. pogona*, but also activated the expression of genes related to polyketide chain biosynthesis (Fig. 1). The overexpression of *bfr* promoted the expression of polyketide synthase genes (Fig. 4d), which may be related to the enhanced iron storage and utilization capacity in *S. pogona* (Fig. 2b). The DEPs identified from quantitative proteomics mainly focused on central carbon metabolism, β-oxidation and amino acid degradation (Fig. 5). The overexpression of *bfr* significantly upregulated the expression of most of the key proteins in these metabolic pathways, while downregulating the key enzymes that control the carbon flux to the pentose phosphate pathway and the TCA cycle. This is conducive to the accumulation of acyl-CoA to provide sufficient precursor materials for the biosynthesis of butenyl-spinosyn (Fig. 6).

These analyses indicated that the overexpression of Bfr caused a substantial increase in the production of butenyl-spinosyns in *S. pogona*, which may be caused by a combination of multiple factors, including enhanced strain resistance, increased supplying acyl-CoA precursors and activation of polyketide synthase genes. Although these phenomena can be explained and verified by macroscopic and extensive metabolic changes, we still do not know how the enhancement of iron utilization affects these metabolic pathways in detail, especially the effect on the butenyl-spinosyn biosynthetic gene cluster. These in-depth mechanisms need to be further explored. In addition, the quantitative proteomics revealed the unique metabolic advantages of ::*bfr*, which suggests that we may be able to optimize the metabolic pathways to further explore the production potential of ::*bfr*, thereby increasing the yield of butenyl-spinosyn.

## Conclusions

**Fig. 8:**
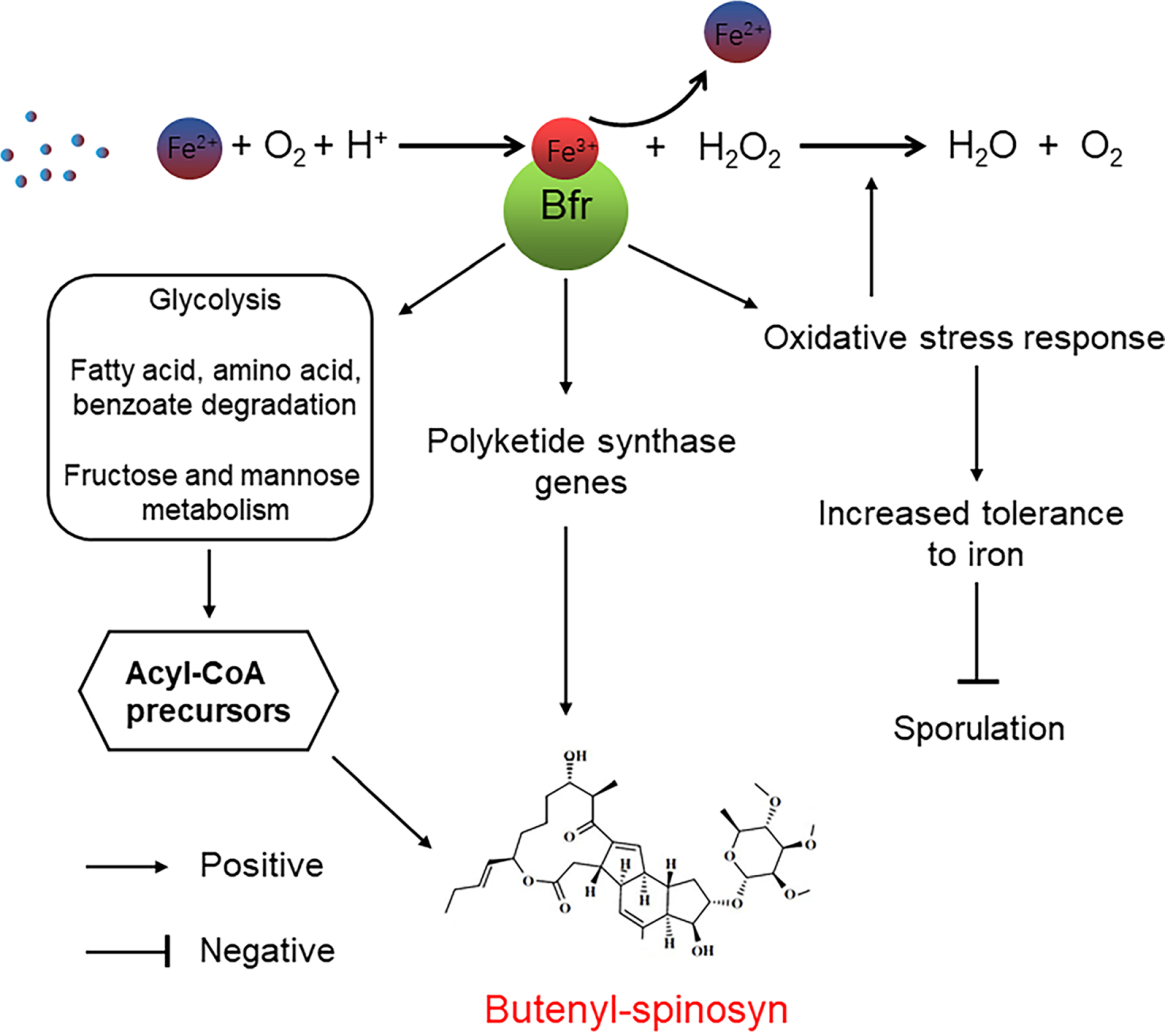
Working model for the coordinated control of growth metabolism and butenyl-spinosyn biosynthesis through Fe and Bfr in *S. pogona*. Fe^2+^ in the environment is absorbed and stored in Bfr in the form of Fe^3+^, which protects cells from iron toxicity followed by release in the form of Fe^2+^ for life activities when needed by cells. Overexpression of *bfr* enhances the ability of strains to store iron, and promotes oxidative stress response to avoid oxidative damage in order to adapt to high iron concentration environments, affects central carbon metabolism and oxidative phosphorylation to synthesize more short-chain acyl-CoA precursors. Together, these factors promote the biosynthesis of butenyl-spinosyn

Collectively, Bfr has an important positive effect on the growth and butenyl-spinosyn biosynthesis in *S. pogona* through regulating iron homeostasis and oxidative stress response (Fig. 8). This research helps us understand the role of iron in microorganism metabolism, and provides references for improving the production of secondary metabolites and strain resistance in actinomycetes.

## Materials and methods

### Plasmids, bacterial strains, media, and growth conditions

The primers, plasmids, and bacterial strains used in this study are listed in Additional file 1: Table S1 and S2. *E. coli* strains were cultured in Luria-Bertani (LB) broth at 37 °C. Unless otherwise specified, *S. pogona* (wt), Δ*bfr* and ::*bfr* strains were all cultivated in complete synthetic medium (CSM: trypticase soy broth 45 g/L, glucose 10 g/L, yeast extract 9 g/L, MgSO_4_ 2.2 g/L) with 20 mL medium per 300 mL bottle, 30 °C, 200 rpm for 48 h. 2 mL of activated bacteria suspension was taken out and added to 50 mL each bottle of synthetic fermentation medium (SFM: glucose 20 g/L, tryptone 4 g/L, yeast extract 4 g/L, MgSO_4_ 0.5 g/L, K_2_HPO_4_ 0.5 g/L, KNO_3_ 1 g/L, FeSO_4_·7H_2_O was added in the corresponding amount as needed, pH 7.5), 30 °C, 200 rpm for 10 days. The R6 medium (BHI 26 g/L, sucrose 200 g/L, dextrin 10 g/L, casamino acids 1 g/L, K_2_SO_4_, 0.1 g/L, FeSO_4_ 0.1 g/L, MgSO_4_ 0.05 g/L, ZnSO_4_ 0.001 g/L, MnCl_2_ 0.001 g/L) was used for the conjugation transfer of *S. pogona* at 30 °C. 1.5% (w/v) agar was added to obtain a solid media. Where applicable, Apramycin (Apra, 50 μg/mL) was added into the above medium for selection.

### Construction of plasmids and verification of recombinant strains

The CRISPR/Cas9 system was used to knock out *bfr* [51]. The sgRNA guide sequence of *bfr* was designed by online tool ZIFIT (http://zifit.partners.org) [52], and fusion with upstream and downstream homologous arms of *bfr* to obtain sgRNA-UHA-DHA. Then the fusion fragment was cloned into pKCcas9dO to obtain pKCcas9dO-sgRNA-UHA-DHA by restriction enzyme digestion and ligation (*Spe* I and *Hind* III) (Additional file 1: Figure S3a). The pOJ260 was used to overexpress *bfr* [53]. *bfr* gene and *kasO*p* promoter was amplified and integrated to obtain *kasO*p*-*bfr*. Then the fusion fragment was cloned into pOJ260 to obtain pOJ260-*kasO*p*-*bfr* by restriction enzyme digestion and ligation (*Xba* I and *Hind* III) (Additional file 1: Figure S3b).

The reconstructed plasmids were transformed into *E. coli* S17 by heat shock, confirmed and introduced into *S. pogona* by conjugation [54] (Additional file 1: Figure S3c, d). Then, the conjugants with an apramycin-resistant phenotype were selected and confirmed by PCR and qRT-PCR to obtain the recombinant strains (Additional file 1: Figure S4).

### Heterologous expression of Bfr and Western blot verification

The *bfr* gene fragment was amplified using primers F-*bfr*/R-*bfr* and cloned into the pET28a vector, and introduced into *E. coli* BL21. Recombinant bacteria were cultured in LB supplemented with 50 μg/mL kanamycin at 37 °C for 4 h, and different concentrations of IPTG (0, 25 μL) were added to induce Bfr protein expression (Additional file 1: Figure S5a). The heterologously expressed protein Bfr was identified by 1D-LC-MS/MS (Additional file 1: Figure S5b), and the anti-Bfr antibody was provided by immunizing BALB/c mice. The Bfr expression level in the wild-type and the mutants was analysed by Western blot [55] (Fig.1d).

### Detection of strain density and butenyl-spinosyn

The wild-type and recombinant strains were cultured in SFM medium for 10 d. Ultraviolet spectrophotometer was used to determine strain density, the wavelength was set to 600 nm. Glass beads were added to the culture medium to avoid the formation of pellets and sediments during the fermentation. The cell suspension was collected every 12 h and diluted to an appropriate concentration for detection during fermentation. The absorbance value (OD_600_) was recorded and the growth curve was drawn.

High performance liquid chromatography 1290 (HPLC 1290) was used to detect the production of butenyl-spinosyn. 1 mL of fermentation broth (10th day) was added to an equal volume of ethyl acetate, extracted in a water bath at 65 °C for 1 h, and centrifuged at 12,000 rpm for 10 min. 500 μL of supernatant was taken out and freeze-dried, then added 50 μL of methanol to fully dissolve, centrifuged at 12,000 rpm for 5 min, the supernatant was taken out for detection. The detection conditions were set as the following: column C18 (AQ12S05-1546WT), 4.5 × 150 mm, 4.5 μm, detection wavelength was set to 250 nm, the sample loading volume was 20 μL. The elution buffer A: 10% (v/v) acetonitrile, buffer B: 90% (v/v) acetonitrile. The following gradient of buffer B was applied: 0 min, 0%; 2 min, 0%; 20 min, 100%; 22 min, 100%; 23 min, 0%; 25 min, 0%. The flow rate was set to 1 mL/min. The peaks of interest were collected and concentrated for LC-MS/MS identification (Additional file 1: Figure S1).

### Insecticidal activity analysis

1 mL of fermentation broth supernatants of different samples were added to 19 mL feed (soy flour 120 g/L, flour 60 g/L, yeast 20 g/L, ethyl Paraben 2 g/L, sorbic acid 1 g/L, Agar 13 g/L, vitamin C 20 tablets, vitamin B_2_ 10 tablets) to detect insecticidal activity against *H. armigera*, mixed well and evenly added to 24-well plates, the number of dead insects was recorded every day for 6 days.

### Total RNA extraction and qRT-PCR analysis

Biomass samples of the wild-type and the recombinant strains cultured in SFM for 4 d were taken out, and total RNA was separated using Total RNA Extractor (Shanghai Sangon Biotech Co., Ltd.) according to the instructions. The RNA concentration and purity were determined by measuring the ratio of OD_260_ to OD_280_. The 7500 Real-Time PCR system instrument (Applied Biosystems, USA) was used to determine the transcription level of the sample. Prime Script^TM^ RT Reagent Kit (Takara) were used for DNase treatment and cDNA synthesis according to the instructions. SYB^®^ Permix Ex Tag^TM^ GC (Takara) was used for qRT-PCR amplification. The primer pairs used in qRT-PCR were listed in Additional file 1: Table S2, and the 16S rRNA gene was used as an internal control to quantify the relative expression level of the samples.

### Protein extraction and SDS-PAGE analysis

Cell suspension of the wild-type and recombinant strains at early stabilization growth phases (4th day) were taken out respectively, and washed 4 times with PBS (prechilled at 4 °C,10 mM, pH 7.8 ) through centrifugation at 10,000 rpm for 10 min at 4 °C, then the cell pellet was resuspended with 200 μL lysozyme (100 mg/mL), adding 600 μL Lysis buffer (2 M thiourea , 50 mM Tris-HCl, 75 mM NaCl, 8 M urea, 4% CHAPS, pH 8.0), 2 μL EDTA (1M) and 5 μL protease inhibitor in each tube, then ultrasonic fragmentation (JY92-ultrasonic cell grinder, Ningbo new Chi biotechnology company) to extract protein (Ultrasound for 3 s, 2 s apart, lasting 99 times). The Bradford method was used to quantify the protein, and the integrity of the sample was checked by SDS-PAGE, then analyzed by LC-MS/MS [56, 57].

### Processing of TMT protein samples and LC-MS/MS analysis

According to the instructions of manufacturer (Thermo Scientific), TMT reagent was used to label 100 μg peptide mixtures of the samples respectively. The labeled peptides were separated using Thermo Scientific's High pH Reversed-Phase Peptide Separation Kit according to the instructions. Q Exactive mass spectrometer combined with Easy nLC (Thermo Scientific) for LC-MS/MS analysis. The samples were loaded onto a reversed-phase capture column (nanoViper C18, 100 μm × 2 cm, Scientific Acclaim PepMap100) connected to a C18 reversed-phase analytical column in 0.1% formic acid (buffer A), buffer B (0.1% formic acid and 84% acetonitrile) was used to separate with a linear gradient, 300 nL/min, positive ion mode. The MASCOT engine (Matrix Science, London, UK; version 2.2) embedded in the Proteome Discoverer 1.4 software was used to search the MS raw data of each sample for identification and quantitative analysis.

### Intracellular iron content detection

The cell pellets of the wild-type and recombinant strains were collected after four days of cultivation and washed 3 times with PBS (10 mM, pH 7.8) at 10,000 rpm for 5 min, and the supernatant was removed. Each tube of sample was added with 200 μL of lysis buffer, incubated at 37 °C for 2 h, and then and then sonicated for 5 min. The detection steps were performed in accordance with the instructions of the Intracellular Iron Colorimetric Assay Kit (Beijing Pulilai Gene Technology Co., Ltd.).

### Determination of intracellular acyl-CoA precursors

The cell pellets of the wild-type and recombinant strains were collected after 2 d, 4 d and 6 d of cultivation, respectively. The freeze-thaw cycle was repeated to destroy the cell wall, then the supernatant was collected at 5000 rpm for 8 min, and the content of acetyl-CoA, malonyl-CoA and methylmalonyl-CoA were measured, respectively. The detection steps were performed in accordance with the instructions of the microorganism acetyl-CoA ELISA Kit, the microorganism malonyl-CoA ELISA Kit, and the microorganism methylmalonyl-CoA ELISA Kit (Shanghai FANKEL Industrial Co., Ltd.)

### Total antioxidant capacity (T-AOC) detection

The cell pellets of the wild-type and recombinant strains were collected after four days of cultivation and washed 3 times with PBS (10 mM, pH 7.8) at 10,000 rpm for 5 min. 1 mL pre-cooled extract was added to each tube sample, then, the cells were disrupted with ultrasound in an ice bath (200 W, ultrasonic 3 s, interval 10 s, repeat 30 times), centrifuged at 10,000 g for 5 min at 4 °C, and placed on ice for testing. The detection steps were performed in accordance with the instructions of the Total Antioxidant Capacity Assay Kit (Beijing Boxbio Science & Technology Co., Ltd.).

### ***Strains resistance to H***_***2***_***O***_***2***_*** exposure***

*S. pogona* grew on CSM solid medium to produce white spores. The spore suspension of about 10^10^ spores/mL was centrifuged at 5000 rpm for 8 min, and resuspended in 5 mL TES (50 mM, pH 8.0). The spore suspension was heat shocked at 50 °C for 10 min, quickly cooled to room temperature in cold water, and an equal volume of double-strength germination medium (CaCl_2_ 1.11 g/L, casamino acid 10 g/L, yeast extract 10 g/L) was added. Then, the spore suspension was incubated with shaking at 37 °C for 3 h. The spore suspension was centrifuged at 4000 rpm for 5 min, the supernatant was removed. The spores were resuspended in water and fully dispersed, inoculated into 5 mL of CSM to OD_450_ of 0.05, and cultured with shaking at 30 °C for 3 h. Then, *S. pogona* cultures were treated with different concentrations of H_2_O_2_ at 30 °C for 30 min. Finally, the cells were collected by centrifugation, diluted to a suitable gradient and plated on CSM agar for counting.

### Statistical analysis

SPSS statistics version 19.0 was used to perform all the statistical analyses. P < 0.05 was considered statistically significant.

## Supplementary Information

**Additional file 1: Table S1.** Strains, plasmids and primers used in this study. Table S2. qRT-PCR primers used in this study. Table S3. Biological insecticidal activity of wt, Δ*bfr* and ::*bfr*. **Figure S1.** LC-MS/MS identification of butenyl-spinosyn. **Figure S2.** Phylogenetic tree analysis of bacterioferritin. **Figure S3.** Construction and recombination schematic diagram of pKCcas9dO-sgRNA-UHA-DHA and pOJ260-*kasO*p*-*bfr*. **Figure S4.** Identification of Δ*bfr* and ::*bfr*. **Figure S5.** Tricine-SDS-PAGE analysis and 1D-LC-MS/MS identification of heterologously expressed protein Bfr. **Figure S6.** Butenyl-spinosyn yield curve of the wild-type and mutants. **Figure S7.** Statistics of total protein and differential protein between the wild-type and mutant strains identified in the quantitative proteome. **Figure S8.** The expression fold change of bus family proteins between *bfr* mutant strains and wild-type strain. **Figure S9.** Verification of the transcription levels of iron homeostasis and oxidative stress-related proteins.

**Additional file 2: Table S4.** DEPs in ::*bfr*.

**Additional file 3: Table S5.** DEPs in Δ*bfr*.

## Data Availability

All data generated or analyzed during this study are included in this published article and its Additional files 1, 2, 3.

## Abbreviations

UHA: Upstream homology arm
DHA: Downstream homologous arm
Bfr: Bacterioferritin
SEM: Scanning electron microscopy
HPLC: High performance liquid chromatography
LC-MS/MS: Liquid chromatography mass spectrometry
DEPs: Differentially expressed proteins
T-AOC: Total antioxidant capacity
SFM: Synthetic fermentation medium
CSM: Complete synthetic medium
