# Supplementary material for: Bacterioferritin: a key iron storage modulator that affects strain growth and butenyl-spinosyn biosynthesis in Saccharopolyspora pogona
Source: Microb Cell Fact. 2021 Aug 14;20:157. doi: 10.1186/s12934-021-01651-x (PMC8364703; doi:10.1186/s12934-021-01651-x)
Supplement: Supplementary file 1 — Additional file 1: Table S1. Strains, plasmids and primers used in this study. Table S2. qRT-PCR primers used in this study. Table S3. Biological insecticidal activity of wt, Δbfr and ::bfr. Figure S1. LC-MS/MS identification of butenyl-spinosyn. Figure S2. Phylogenetic tree analysis of bacterioferritin. Figure S3. Construction and recombination schematic diagram of pKCcas9dO-sgRNA-UHA-DHA and pOJ260-kasOp*-bfr. Figure S4. Identification of Δbfr and ::bfr. Figure S5. Tricine-SDS-PAGE analysis and 1D-LC-MS/MS identification of heterologously expressed protein Bfr. Figure S6. Butenyl-spinosyn yield curve of the wild-type and mutants. Figure S7. Statistics of total protein and differential protein between the wild-type and mutant strains identified in the quantitative proteome. Figure S8. The expression fold change of bus family proteins between bfr mutant strains and wild-type strain. Figure S9. Verification of the transcription levels of iron homeostasis and oxidative stress-related proteins. [file 12934_2021_1651_MOESM1_ESM.doc]

**Bacterioferritin: a key iron storage modulator that affects strain growth and butenyl-spinosyn biosynthesis in *Saccharopolyspora pogona***

Jianli Tang^#^, Zirong Zhu^#^, Haocheng He, Zhudong Liu, Ziyuan Xia, Jianming Chen, Jinjuan Hu, Li Cao, Jie Rang, Ling Shuai, Yang Liu, Yunjun Sun, Xuezhi Ding, Shengbiao Hu, Liqiu Xia*

Hunan Provincial Key Laboratory for Microbial Molecular Biology, State Key Laboratory of Developmental Biology of Freshwater Fish, College of Life Science, Hunan Normal University, Changsha, China.

*Correspondence to: Liqiu Xia, Hunan Provincial Key Laboratory for Microbial

Molecular Biology, State Key Laboratory of Development Biology of Freshwater

Fish, College of Life Science, Hunan Normal University, Lushan Road 36,

Changsha 410081, China. Tel/ Fax: +86 073188872298.

E-mail address: [xialq@hunnu.edu.cn](mailto:xialq@hunnu.edu.cn)

Jianli Tang^#^ and Zirong Zhu^#^ contribute equally to this work.

**Table S1. Strains, plasmids and primers used in this study**

| **Strains** | **Relative description** | **Sources** |
| --- | --- | --- |
| *E. coli* Top10 | Containing pUC57-Amp- *kasOp** | Lab store |
| *E. coli* DH5α | Host for general cloning | Lab store |
| *E. coli* S17 | Donor strains for conjugation | Lab store |
| *E. coli* BL21 | Heterologous expression host | Lab store |
| *S. pogona* | The producer strains of butenyl-spinosyn | Lab store |
| *E. coli* BL21-*bfr* | Containing pET28a-Kan-*bfr* | This work |
| ::*bfr* | *S. pogona* harboring pOJ260-*kasOp*-bfr* | This work |
| Δ*bfr* | *bfr* knockout mutant of *S. pogona* | This work |
| **Plasmids** |  |  |
| pOJ260 | *E. coli*-cloning vector, containing pUC18 replicon, oriT, Apra^R^ | Lab store |
| pET28a | Protein heterologous expression vector | Lab store |
| pUC57-Amp-*kasOp** | Containing *kasOp** promoter sequence | Lab store |
| pKCcas9dO | E. coli-cloning vector, containing Scocas9, oriT, Apr^R^ | Lab store |
| pET28a-Kan-*bfr* | *bfr* inserted into pET28a by *EcoR* I and *Hin*d III | This work |
| pOJ260-*kasOp**-*bfr* | *kasOp**-*bfr* inserted into pOJ260 by *Xba* I and *Hin*d III | This work |
| pKCcas9d-sgRNA-UHA-DHA | sgRNA-UHA-DHA inserted into pKCcas9dO by *Hin*d III and *Spe* I | This work |
| **PCR Primers** | Sequence (5′→ 3′) |  |
| sgRNA-F | TTGG**ACTAGT**GCCAAGACCGCCCGCGAGGAGTTTTAGAGCTAGAAA | This work |
| sgRNA-R | CTCAAAAAAAGCACCGACTCGG | This work |
| *bfr*-up-F | AGTGGCACCGAGTCGGTGCTTTTTTTGAGCGACCTGATCTACGCCTACCA | This work |
| *bfr-*up-R | CTCCTCGACCTGCTCCTTCAGGAACCACTGGCTGCTCCATTTAGCGAAGG | This work |
| *bfr*-down-F | CAGTGGTTCCTGAAGGAGCA | This work |
| *bfr-*down-R | CCC**AAGCTT**CAAGACGACCTACGGCAAACT | This work |
| *kasOp**-F | TGC**TCTAGA**TGTTCACATTCGAACGGTCTC | This work |
| *kasOp**-R | AACTCCCCCAGTCCTGCAC | This work |
| *bfr*-F | GACAGCGTGCAGGACTGGGGGAGTTATGGCTGTCACCGCGAAGATT | This work |
| *bfr*-R | CCC**AAGCTT**CCGTTCCTGTCGTTCCTACC | This work |
| Apr-F | GTCCAATACGAATGGCGAAAAGC | This work |
| Apr-R | ATAACATTCTTCGCATCCCGCC | This work |
| F-*bfr* | CCG**GAATTC**ATGGCTGTCACCGCGAAGATT | This work |
| R-*bfr* | CCC**AAGCTT**CCGTTCCTGTCGTTCCTACC | This work |

Note: Restriction enzyme sites were bold, overlapping sequences were underlined, and sgRNA sequences were marked in red.

**Table S2. qRT-PCR Primers used in this study**

|  | **Relative description** | **Sources** |
| --- | --- | --- |
| **qRT-PCR Primers** | Sequence (5′→ 3′) | This work |
| 16s rRNA-F | CGTCAGCTCGTGTCGTGAGA | This work |
| 16s rRNA-R | GTGAAGCCCTGGGCATAAGG | This work |
| *bfr*-F_qRT_ | ATGGCTGTCACCGCGAAGAT | This work |
| *bfr*-R_qRT_ | GTAGAAGTACGCGGCCAGCC | This work |
| *sigF-*F | GCAGATGACCAGCGTGAT | This work |
| *sigF-*R | TAGAGGGTGACCACCACG | This work |
| *whiB-*F | GCGACGGACGAAGAACAGG | This work |
| *whiB-*R | CAGCGCGTACTCCAGGCAT | This work |
| *whiA-*F | CCGACGGGCTGAGGTTTC | This work |
| *whiA-*R | GTGCCCGAACAGCTCGTG | This work |
| *ssgA*-F | CGAGGGCGACGTGACGAT | This work |
| *ssgA*-R | AGGTTCTCGTTGCCAGGCAC | This work |
| *busA*-F | ACCAACGACGATGAACACGC | This work |
| *busA*-R | GCAACCTCCCTGGATTACGG | This work |
| *busB*-F | CGCTTGGCTCAGGTGTCGT | This work |
| *busB*-R | GAAGACGGATGTTCGTGACCC | This work |
| *busC*-F | CAGGGGAACAGGCGAAAT | This work |
| *busC*-R | GCGTAGACACGGTTGTTGAG | This work |
| *busD*-F | GCAGGGCATTCCGTCCATT | This work |
| *busD*-R | ACCGAGCAGGACTTCCAACG | This work |
| *busE*-F | GCCTCCACAGCATCCACATC | This work |
| *busE*-R | TGTCGGATGCCCGTCGTA | This work |
| *fer*-F | ATCGAGGAATGCCCTGTCG | This work |
| *fer*-R | CCACGTTCGCCTTGGTGTAG | This work |
| *trxB*-F | CCAGGACATCGCCGTCATCG | This work |
| *trxB*-R | GCCTTGTTGGTCAGCCACTT | This work |
| *katG*-F | GACCTGGGCACCAAGTGGAA | This work |
| *katG*-R | CGTCGTCGCTCGCATAAACC | This work |
| *sodN*-F | CCTGTGGGTGCTGTGGAC | This work |
| *sodN*-R | TCGATCTCGGCGATGTAGT | This work |

***Table S3. Biological insecticidal activity of wt, Δbfr and ::bfr***

| **Strains** | **LT_50_ (d)** |  | **95% Confidence interval** |
| --- | --- | --- | --- |
| wt | 5.919 |  | 5.400-6.700 |
| Δ*bfr* | 7.445 |  | 6.591-9.043 |
| ::*bfr* | 3.808 |  | 3.551-4.073 |

**
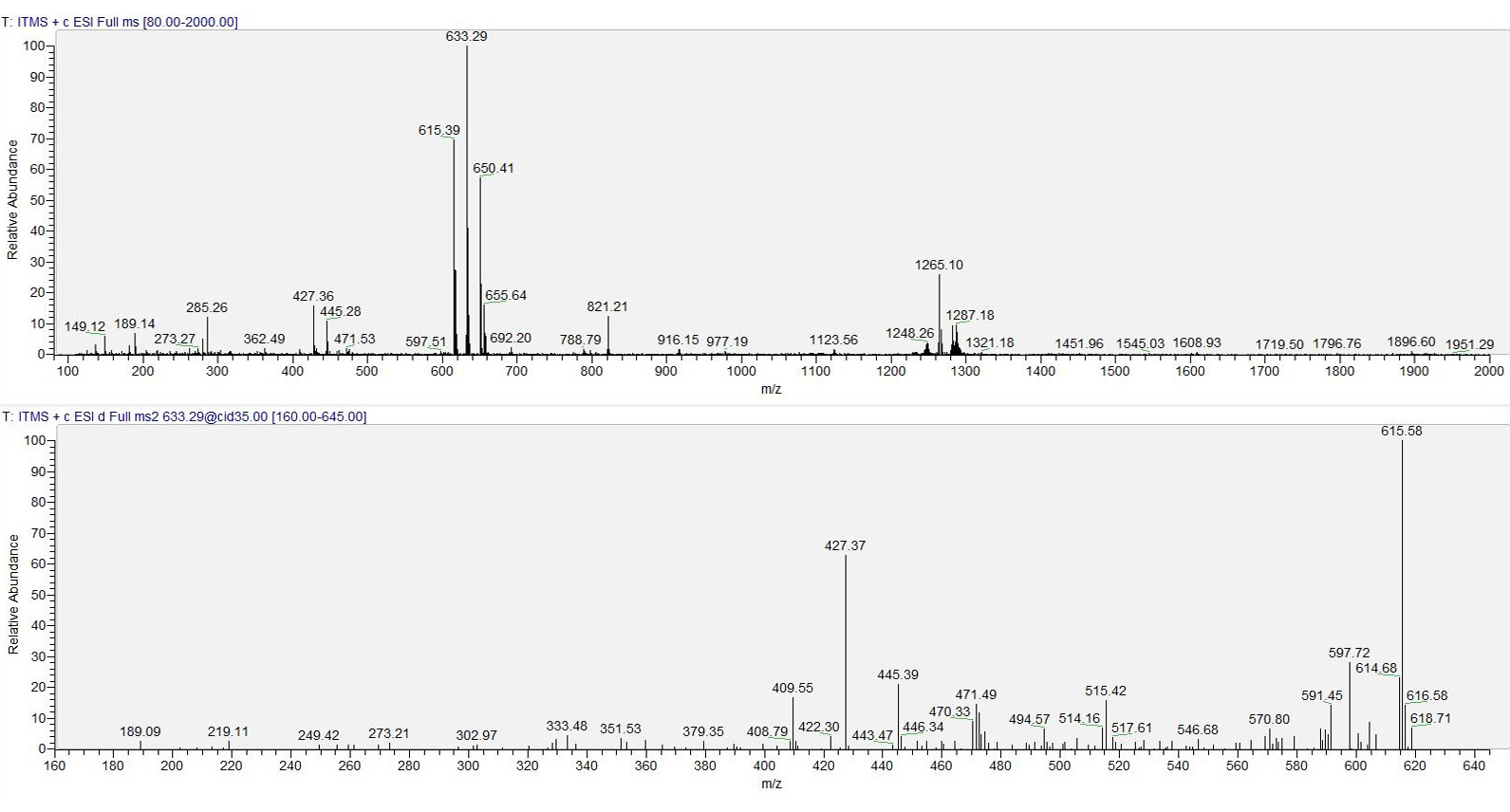
Figure S1. Mass spectrum identification of butenyl-spinosyns.** MS identification results showed that MS parent ion [M+H]^+^ = 633.29 contained a trimethylrhamnose fragment of 189.09 molecular mass, which was confirmed as a butenyl-spinosyn component.


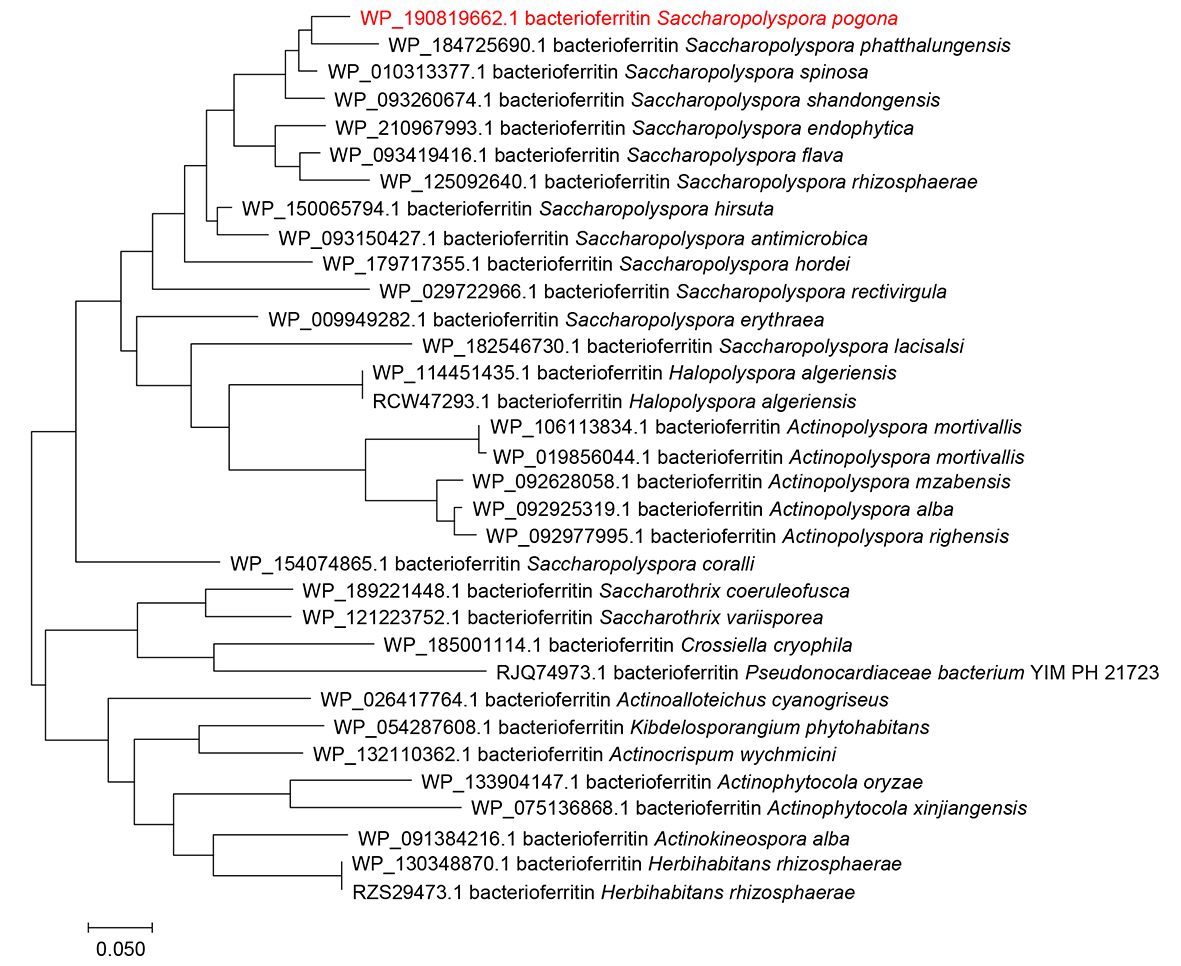


**Figure S2. Phylogenetic tree analysis of bacterioferritin (red).** MEGA7.0.26 was used to construct the tree by the neighbor-joining method based on Bfr protein sequence.

**
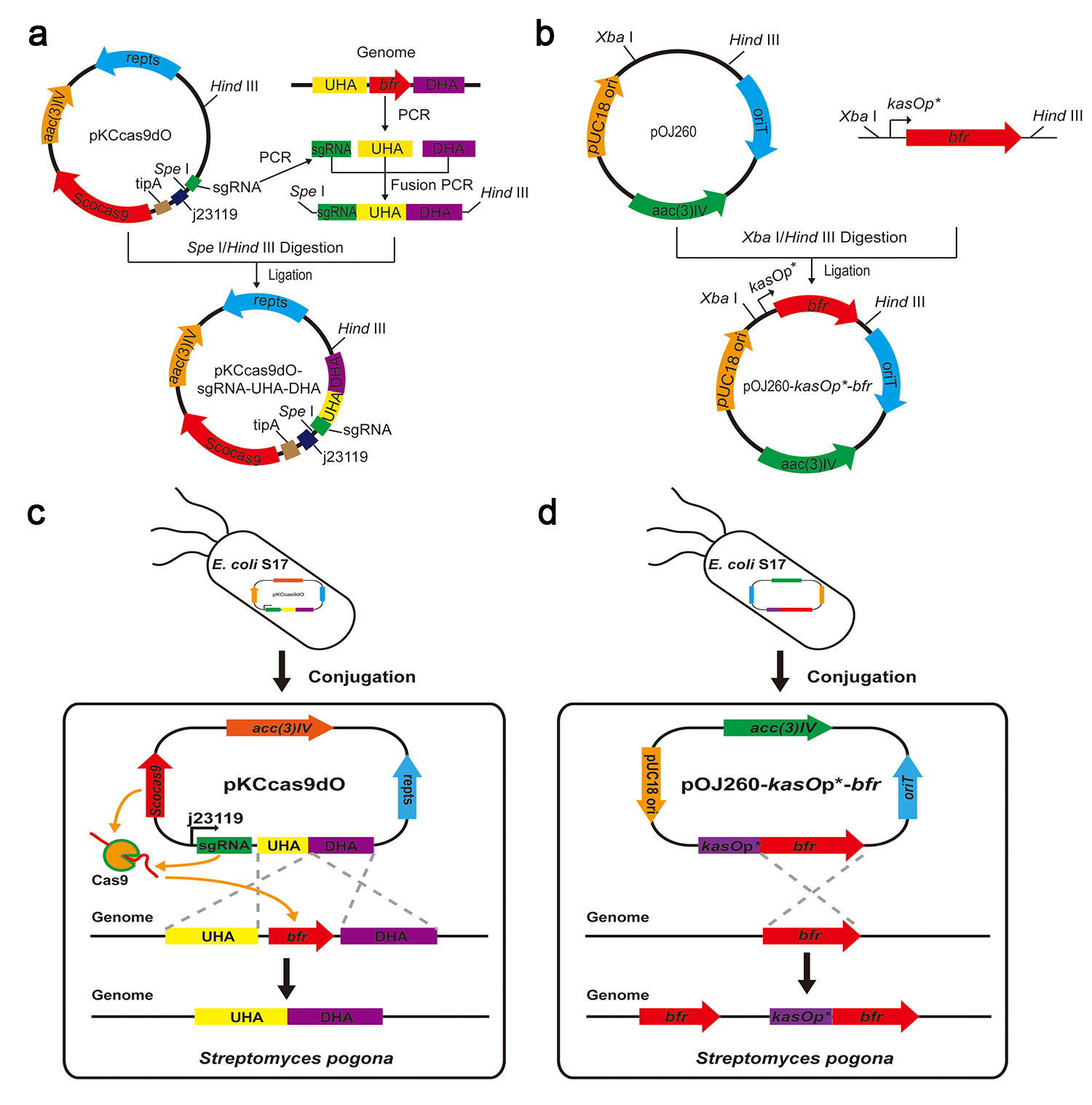
**

**Figure S3.** **Construction and recombination schematic diagram of** **pKCcas9dO- sgRNA-UHA-DHA and pOJ260-*kasOp***-bfr*.** **a** Construction of plasmid pKCcas9dO-sgRNA-UHA-DHA. **b** Construction of plasmid pOJ260- *kasOp*-bfr*. **c** Recombination schematic diagram of pKCcas9dO-sgRNA-UHA-DHA. **d** Recombination schematic diagram of pOJ260-*kasO*p**-bfr.*


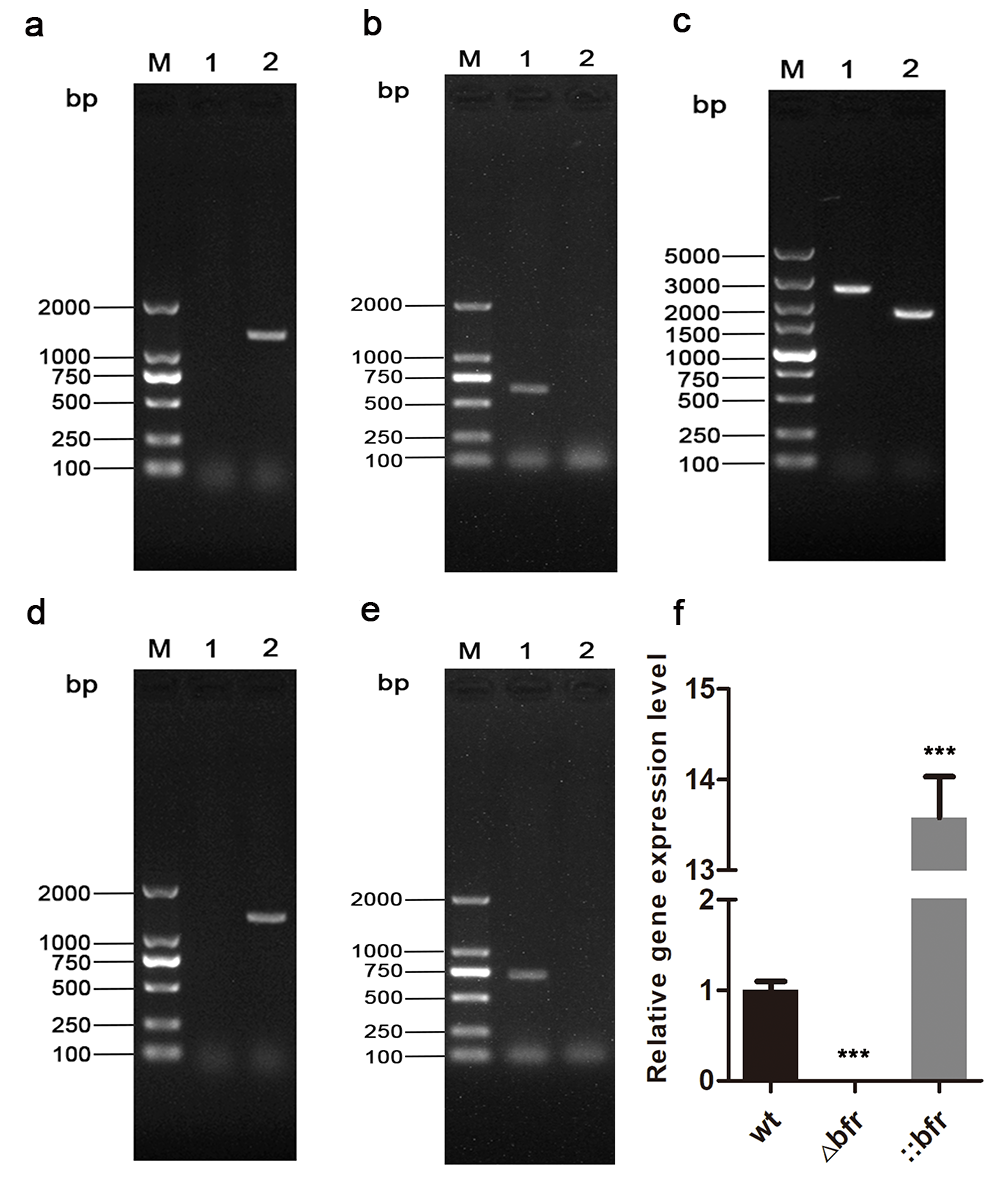


**Figure S4.** **Identification of Δ*bfr* and ::*bfr*.** **a** PCR amplification of *aac(3)*IV gene in *S. pogona and* Δ*bfr*. M: DL 2000 DNA marker; 1: PCR products of *S. pogona* with primer pair Apr-F/Apr-R; 2: PCR products of Δ*bfr* with primer pair Apr-F/Apr-R. **b** Identification of *bfr* gene in *S. pogona and* Δ*bfr.* M: DL 2000 DNA marker; 1: PCR products of *S. pogona* with primers *bfr*-F/*bfr*-R; 2: PCR products of Δ*bfr* with primers *bfr*-F/*bfr*-R. **c** PCR amplification from UHA to DHA in *S. pogona and* Δ*bfr.* M: DL 5000 DNA marker; 1: PCR products of *S. pogona* with primers *bfr*-up-F/*bfr-*down-R; 2: PCR products of Δ*bfr* with primers *bfr*-up-F/*bfr-*down-R. **d** PCR amplification of *aac(3)*IV gene in *S. pogona and* ::*bfr*. M: DL 2000 DNA marker; 1: PCR products of *S. pogona* with primer pair Apr-F/Apr-R; 2: PCR products of ::*bfr* with primer pair Apr-F/Apr-R. **e** Identification of *kasO*p**-bfr* fragment in *S. pogona and* ::*bfr.* M: DL 2000 DNA marker; 1: PCR products of ::*bfr* with primers *kasO*p*-F/*bfr*-R; 2: PCR products of *S. pogona* with primers *kasO*p*-F/*bfr*-R. **f** qRT-PCR verified the *bfr* recombinant strains. 16S rRNA served as the normalization control. Averages from three biological replicates are shown. Error bars represent the standard deviation of the mean. *, **and *** indicate P<0.05, P<0.01 and P<0.005, respectively, compared to the wild-type under the same conditions.


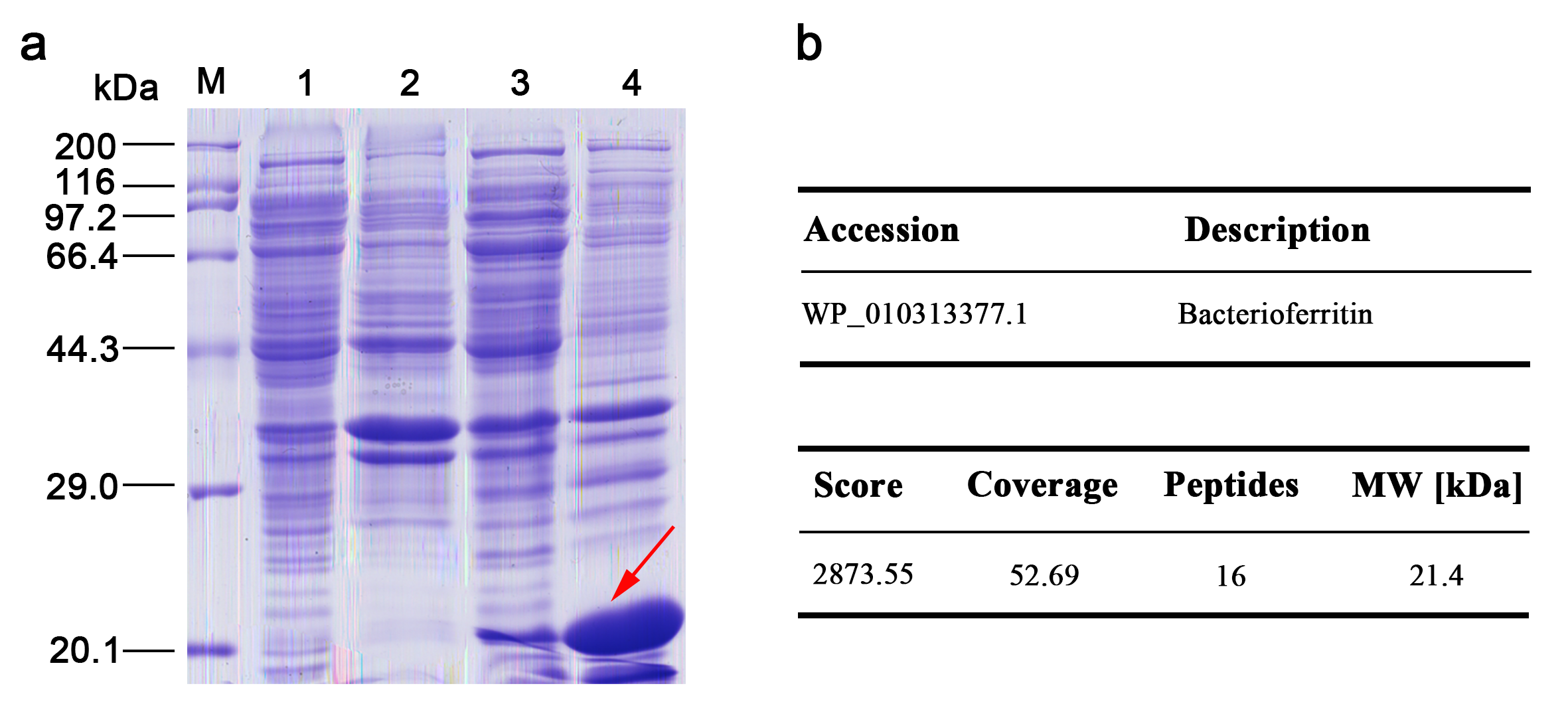


**Figure S5.** **Tricine**-**SDS-PAGE analysis and 1D-LC-MS/MS identification of heterologously expressed protein Bfr. a** Coomassie Brilliant Blue staining of Tricine**-**SDS-PAGE showing heterologous protein Bfr expressed in the supernatants of *E. coli* BL21 bearing recombinant plasmid after IPTG induction and ultrasonication. M: 200 kDa protein marker; 1, 2: Precipitation and supernatant samples from *E. coli* BL21 contained the recombinant plasmid as a negative control without IPTG, 37 ℃ for 4 h. 3, 4: Precipitation and supernatant samples from *E. coli* BL21 contained the recombinant plasmid, 25 uL IPTG, 37 ℃ for 4 h. **b** 1D-LC-MS/MS identification of heterologously expressed protein Bfr.


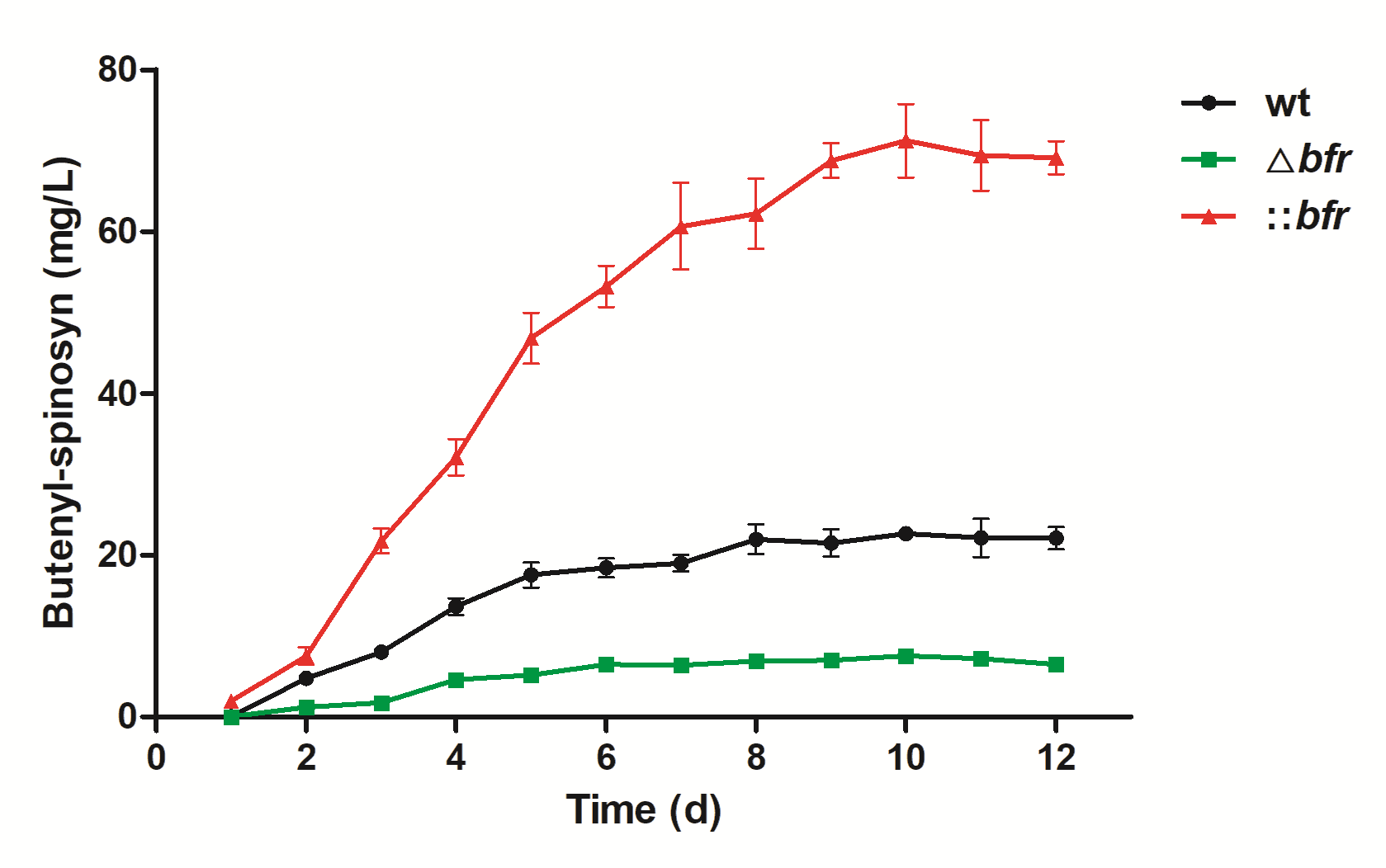


**Figure S6. Butenyl-spinosyn yield curve of the wild-type and mutants.** The production of butenyl-spinosyn reached its maximum on the 10th day.


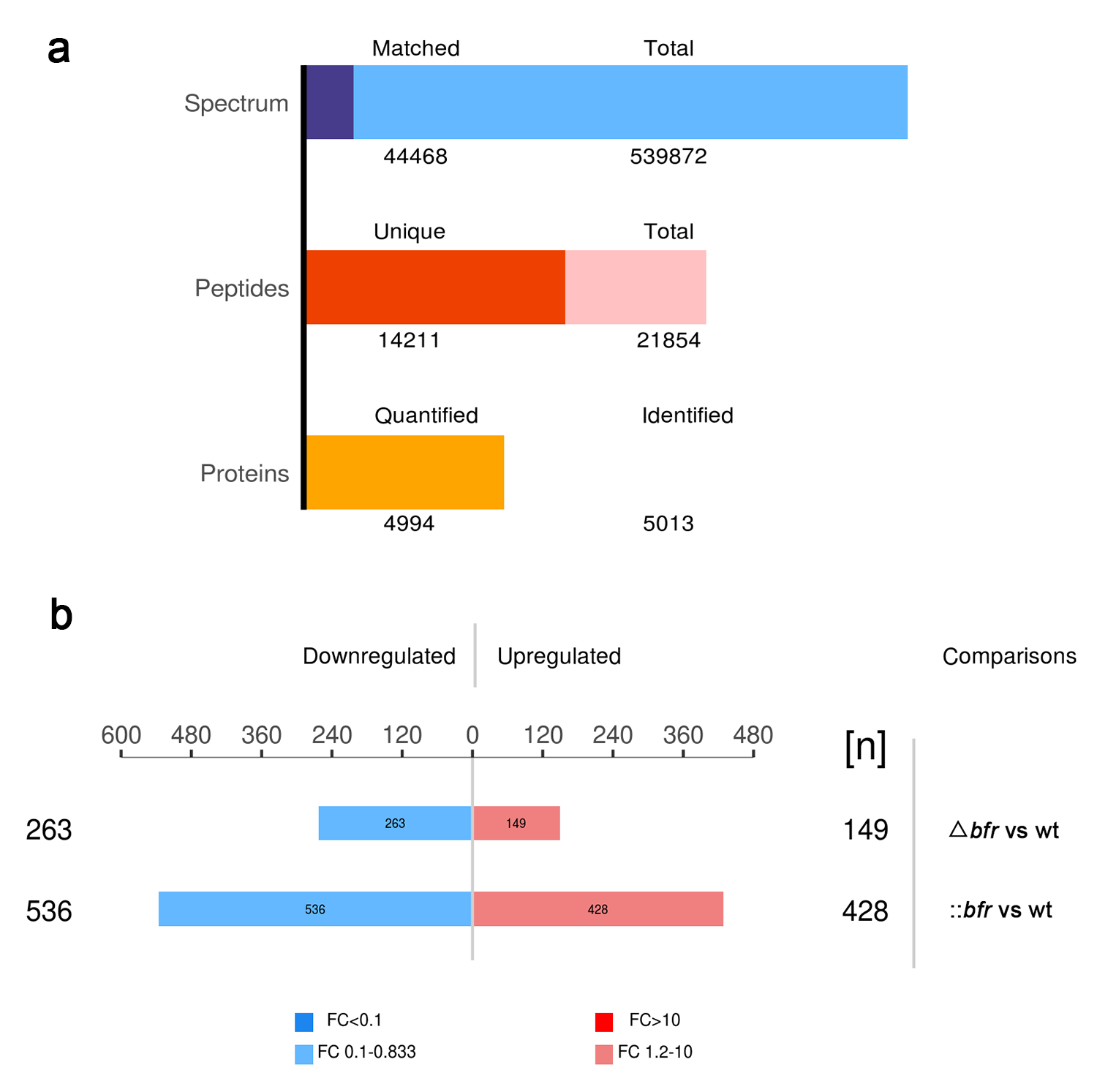


**Figure S7.** **Statistics of total protein and differential protein between the wild-type and mutant strains identified in the quantitative proteome.** **a** A total of 5013 proteins were identified, of which 4994 were quantified. **b** In the significant difference proteins screening, the expression fold change > 1.2 or < 0.833 and P < 0.05 was used as the standard to obtain the comparison group upregulation and downregulation of protein number.


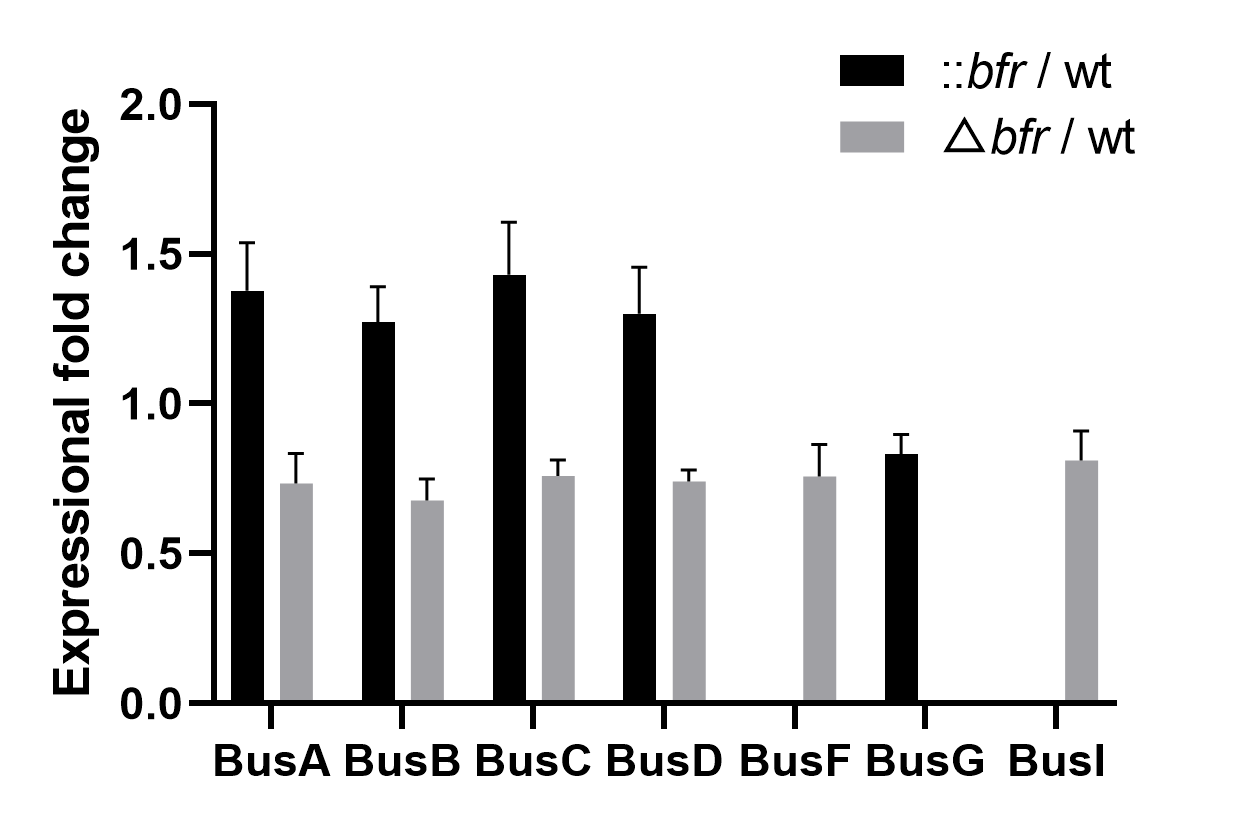


**Figure S8.** **The expression fold change of bus family proteins between *bfr* mutant strains and wild-type strain.** A limited number of bus proteins with significant differences were detected in quantitative proteomic analysis. The expression levels of BusA (Accession: A0A2N3Y5Z2), BusB (Accession: A0A2N3Y616), BusC (Accession: Q9ALM4), BusD (Accession: A0A2N3Y630) and BusG (Accession: Q4JHR2) in ::*bfr* were 1.37-, 1.27-, 1.42-, 1.30-, and 0.83-fold higher than those in the wild-type strain, respectively. The expression levels of BusA, BusB, BusC, BusD, BusF (Accession: Q4JHR3) and BusI (Accession: Q4JHR0) in Δ*bfr* were 0.73, 0.68, 0.76, 0.74, 0.76, and 0.81 times that of the wild-type strain, respectively. This statistical result is based on all DEPs data (fold change > 1.2 or < 0.833, P < 0.05).


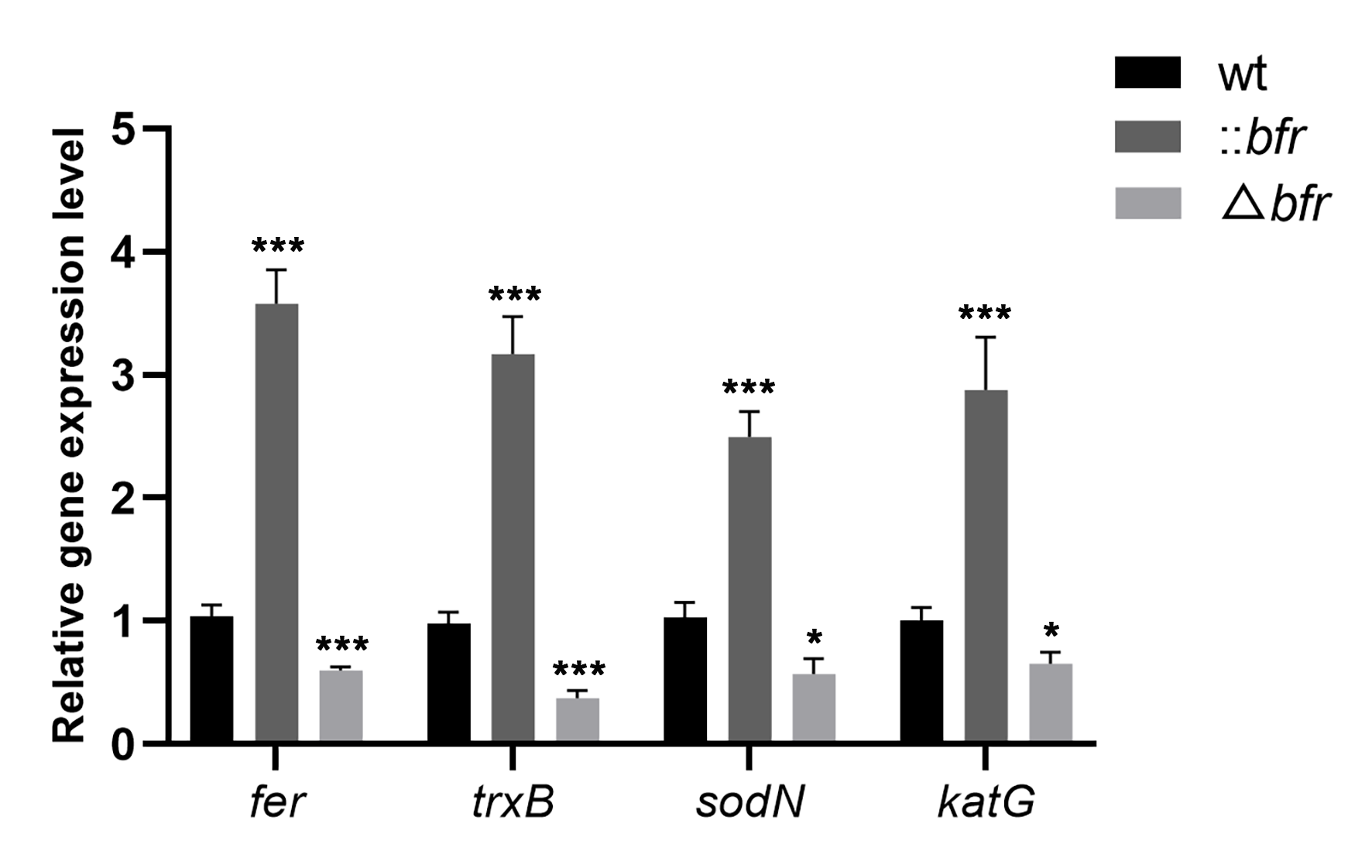


**Figure S9.** **Verification of the transcription levels of iron homeostasis and oxidative stress-related proteins.** The expression levels of these genes (*fer*, *trxB*, *sodN*, and *katG*) in ::*bfr* were 3.45-, 3.25-, 2.43-, and 2.87-fold higher than those in the wild-type strain, respectively. The expression levels of them in Δ*bfr* were 0.58, 0.38, 0.56, and 0.65 times that of the wild-type strain, respectively. 16S rRNA served as the normalization control. Averages from three biological replicates are shown. Error bars represent the standard deviation of the mean. *, **and *** indicate P < 0.05, P < 0.01 and P < 0.005, respectively, compared to the wild-type under the same conditions.
